# Optimizing vaccine site locations while considering travel inconvenience and public health outcomes

**DOI:** 10.1007/s10729-026-09778-2

**Published:** 2026-07-13

**Authors:** Suyanpeng Zhang, Sze-Chuan Suen, Han Yu, Maged Dessouky, Fernando Ordonez

**Affiliations:** 1 Daniel J. Epstein Department of Industrial and Systems Engineering, Viterbi School of Engineering, University of Southern California, 3715 McClintock Ave, 90089 Los Angeles, CA USA; 2 Department of Industrial Engineering, Universidad de Chile, Beauchef 850, 8370451 Santiago, Región Metropolitana Chile

**Keywords:** Vaccine sites, Vaccine allocation, COVID-19, Travel inconvenience

## Abstract

During the COVID-19 pandemic, selecting vaccination sites and allocating limited doses required balancing accessibility, disease control, and fairness. We formulate a multi-objective mixed-integer linear programming model that jointly determines the locations of *mega-sites* and allocates vaccine doses while explicitly incorporating travel inconvenience, disease dynamics, and equitable distribution. The model incorporates commuting patterns from both residential and workplace origins to more accurately capture population mobility, and employs a tractable objective formulation that proxies key public health goals, enabling efficient and equitable mass vaccination planning. Compared with the solution empirically used in Los Angeles County in 2020, we recommend more dispersed *mega-site* locations that result in a 26% reduction in travel inconvenience and avert an additional 200 infections.

**Supplementary Information:**

The online version contains supplementary material available at https://doi.org/10.1007/s10729-026-09778-2.

## Highlights

- We formulate an optimization model to select vaccination sites and assign individuals for vaccination that considers commuting patterns and public health outcomes.
- We devise a tractable expression for incorporating health outcomes into the optimization objective that takes into account vaccination targets, prioritization over areas with a higher risk of becoming infectious, and equitable distribution.
- We run the optimization model with empirical COVID-19 and traffic data in Los Angeles County. We find that with a proper setup, both travel inconvenience and public health outcomes can be improved from the empirically used solution.

## Introduction

As of April, 2022, nearly 500 million COVID-19 cases had been reported worldwide, resulting in approximately 6 million confirmed deaths globally [1]. It imposed significant burdens on both public health and the economy for the entire population [2]. Months after the COVID-19 outbreak, vaccines were developed to control the spread of COVID-19 as well as prevent severe illness [3, 4].

When COVID-19 vaccines first became available and distribution through local pharmacies had not yet been established, practitioners often had to set up mass vaccination facilities (hereafter referred to as *mega-site*) and allocate doses in a way that quickly and efficiently reduced the disease burden.

The locations of *mega-sites* should be strategically chosen to prioritize vaccination for populations in high-risk infection areas, thereby reducing the disease’s spread. Additionally, these sites should be placed to reduce the overall travel inconvenience for individuals, since these additional time costs could present an additional barrier to vaccine access. However, while traditional literature have considered facility location or resource allocation problems, vaccination site allocation requires incorporating additional factors that are often missing from traditional formulations.

Prior work has shown that shorter trips that require less travel inconvenience and less time off work may lead to greater vaccine access [5–7]. Strategically placed *mega-sites* may therefore improve vaccination coverage. However, prior work on healthcare facility location and health transportation typically focuses on transportation costs, where transportation costs are typically measured as the distance from the home to the medical service center. This does not consider patients’ commuting patterns, which may mean that the travel inconvenience in the traditional literature may not reflect the actual inconvenience to vaccine sites.

Exploiting commuting patterns to place health facilities may improve both problem realism and allow for better transportation solutions. Identifying realistic optimal locations for vaccination requires considering how vaccination would be integrated into a patient’s daily life. In particular, travel inconveniences can occur in various situations for commuters, such as when commuting to a vaccination site while on the way to or from work, or when traveling from work or home to a vaccine site and then returning to work or home afterward. Commuting patterns must therefore be integrated into decisions for optimal placement of *mega-site* locations, as individuals may travel to vaccine sites before or after work, and vaccine sites can be conveniently placed next to workplaces or close to home.

It is also important to consider public health outcomes in addition to travel inconvenience when choosing vaccine *mega-site* locations. Residents from a high infection risk area should be able to be assigned to a convenient vaccination *mega-site* with low travel inconvenience to improve health outcomes such as the number of infections averted [8]. To do this, it is critical to integrate the disease dynamics into decision-making on which group should be prioritized for vaccination in order to mitigate infections.

While many works have studied vaccine allocation and facility location problems for infectious disease, we here take a novel approach by integrating travel inconvenience, public health outcomes, and preferences for equitable accessibility into an optimization model to assist public health decisions around determining vaccine supply locations and allocating vaccines.

### Literature review

Prior works have studied vaccination allocation problems as well as locations of vaccination sites. However, their models do not jointly incorporate travel inconvenience and public health outcomes into the optimization objective when both resource allocation and vaccine site location are decision variables. In our review, we found one study that considers public health outcomes in the context of vaccine site location but does not consider travel inconvenience [9]. Resource allocation models typically incorporate public health outcomes, but none of them consider the location of vaccination sites, as these are predetermined in their settings. Additionally, none of the papers we reviewed that account for travel inconvenience incorporate commuting patterns. In our experiment, we demonstrate that including commuting patterns can significantly change the resulting solution. Our paper is the only one, to the best of our knowledge, that integrates decisions on both resource allocation and facility location while accounting for travel inconvenience from commuting patterns, public health outcomes, and equity measures in the objective. This is important because effective and equitable vaccine distribution depends not only on where resources are sent, but also on how easily people can access them and how those decisions influence disease spread across different populations. Next, we review the literature on vaccination center (facility) location and resource allocation models, highlighting key related studies and clarifying how our approach differs from the existing work.

#### Facility location problems in healthcare

There is a rich literature on facility location problems in healthcare. Traditional research on the location of healthcare facilities has primarily focused on coverage-based and assignment-based models [10]. This work is related to both aspects as it addresses both by minimizing travel inconvenience and ensuring sufficient coverage in each region to achieve herd immunity as quickly as possible. The main goal of the coverage-based facility location problem is to ensure that all or most demand points are within a certain distance or time from at least one facility [11–20]. Few have studied demand coverage problems in the contexts of choosing the vaccination site [18–20]. Polo et al. (2015)[19] proposed a bi-objective model that maximizes coverage while minimizing the distance to vaccination centers. However, their approach does not incorporate disease dynamics like much of the healthcare facility location literature [10]. Lim et al. (2016) [18] and Srivastava et al. (2021) [20] addressed vaccination center location problems by maximizing demand coverage, but did not aim to minimize inconvenience costs or incorporate disease dynamics. Notably, Lim et al. (2016) [18] introduced a formulation that accounts for vaccine hesitancy as a function of distance to the vaccination center, a feature we also incorporate in our model.

Assignment-type facility location models, on the other hand, minimize the cost or distance of assigning each demand point to a facility [9, 19, 21–26]. Kumar et al. (2023) [24] and Leithauser et al. (2021) [25] proposed to minimize traveler inconvenience along with operational costs, such as the number of vaccination centers and associated staffing expenses. However, they did not incorporate disease dynamics, equity, or regional coverage. Lusiantoro et al. (2022) [26] and Polo et al. (2015) [19] considered both travel inconvenience and coverage, making them closely related to our work. Nonetheless, our approach is distinct in that it explicitly integrates disease dynamics into the objective function, using region-specific coverage targets that reflect local epidemic conditions. To the best of our knowledge, only two studies—Cao et al. (2023) [23] and Bertsimas et al. (2021) [9]—have considered infection risk in facility location problems while also addressing travel inconvenience. Cao et al. (2023) [23] focused on locating disposal facilities during COVID-19 with the goal of minimizing infection risk, but their model does not involve vaccination needs and does not account for heterogeneity in commuting patterns. Bertsimas et al. (2021) [9] allocated 100 mass vaccination sites across 51 U.S. states, but due to the large-scale planning scope, the commuting patterns cannot be considered, and they do not consider travel inconvenience as an objective. They also made the simplifying assumption that all individuals living in the same population center go to the exact same vaccine site. While this may be reasonable at a national scale, it is overly simplistic in settings like Los Angeles County or other cities where multiple vaccine sites are available. There are also many assignment-based facility location models that focus on reducing operational costs [27–29], which do not consider health dynamics and commuting patterns in the objective. A broad range of general facility location models exists, which we do not cover comprehensively in this review [30–38]. While these models consider factors beyond travel inconvenience, they are not directly applicable to healthcare facility location problems, as they do not incorporate infection risks or disease dynamics. Therefore, compared to the existing literature on facility location problems, our work is the only one that incorporates both disease dynamics and travel inconvenience derived from commuting patterns. This ensures vaccine site placement and vaccine allocation decisions are not only epidemiologically effective but also accessible to the population. For a more comprehensive overview of general facility location models, we refer readers to [39].

#### Health outcomes in resource allocation

A separate body of literature has considered health outcomes in resource allocation problems, but these studies focus solely on allocating resources rather than determining the locations of distribution centers [40–69]. For example, Buhat et al. (2021) [48] developed a linear programming formulation for optimizing vaccine allocation in the Philippines. A prioritization score is assigned to each region and is computed based on the local basic reproduction number. These studies concentrate on the allocation of resources and their impact on public health outcomes. However, they do not take into account travel inconvenience, as the placement of vaccination sites is either predetermined or not a focus of their analyses. Similarly, Orgut et al. (2023) [63] minimized travel inconvenience under the assumption of pre-determined vaccination site locations. Other studies primarily focus on health outcomes or healthcare costs, which do not consider travel inconvenience.

Among studies that incorporate public health outcomes or disease dynamics, most adopt discrete-time compartmental models such as SIR or its variants [9, 54, 59, 61, 64, 65]. For instance, Rao and Brandeau (2021) [64] used a discretized SIR model and based their objective on its output. Bertsimas et al. (2021) [9] proposed a more complex model with multiple compartments beyond SIR. However, such models face practical challenges: small discretization leads to high computational cost, while large discretization sacrifices accuracy. Additionally, complex models like that of Bertsimas et al. (2021) [9] can be difficult to calibrate in practice. On the other hand, Jadidi et al. (2021) [58] proposed a herd immunity function, which is similar to ours. However, their model accounts only for disease dynamics, without incorporating equity or travel inconvenience. In our work, we incorporate prioritization scores and a herd immunity target as proxies for disease dynamics. Our numerical results show that this approach effectively captures key public health outcomes while reducing model complexity.

This study also considers regional vaccine allocation prioritization in our formulation. Geographical prioritization strategies for vaccination allocation have can be highly efficient in safeguarding groups at high risk of becoming infected [70–78], and numerous techniques have been proposed to geographically prioritize groups at high risk of infection [73, 79, 80]. In this paper, we use traffic flow to proxy for infection risk from increased social contacts as the mobility patterns are strongly correlated with decreased COVID-19 case growth rates [81], and we prioritize vaccination accessibility for regions that have greater transportation activity.

### Contributions

This study makes several contributions. Although related problems have been studied extensively, our work is, to the best of our knowledge, the only one that integrates resource allocation and facility location decisions while incorporating travel inconvenience from commuting patterns, public health outcomes, and equity considerations. Also, we devise a tractable expression for incorporating health outcomes into the optimization objective that takes into account vaccination coverage targets, prioritization over areas with a higher risk of becoming infectious, and equitable distribution. Specifically, we propose a way to compute coverage targets (to reach herd immunity) for different geographical areas. We also incorporate prioritization scores that are shown in the numerical experiment to be representative of infection risks. We additionally incorporate preferences around the equitable distribution of vaccinations into the objective function to ensure smoothness in vaccination uptake across different regions. We demonstrate that including inequity scores (measured as the gap between the most and least covered regions) in our numeric experiment improves the public health outcome (number of infections averted). Moreover, we run the optimization model with empirical COVID-19 and traffic data in LAC, the most populous county in the U.S. and an epicenter in the COVID-19 pandemic, and we compare outcomes using the optimal *mega-site* locations with those used in LAC 2020. We demonstrate that with an appropriate setup, it is possible to improve both travel convenience and public health outcomes compared to the empirically implemented solution. We also incorporate vaccine hesitancy in a sensitivity analysis, which has been considered in related studies [18], and find that our framework continues to yield improvements in both dimensions. These findings offer valuable insights for policymakers when planning large-scale vaccination sites to control transmissible diseases during the early phases of vaccine rollout.

The remainder of this paper is organized as follows: we present the problem setup, the optimization formulation, and output evaluation in Section 2. The numerical example is shown in Section 3. In Section 4, we conclude.

## Model formulation and evaluation

In this section, we present an optimization formulation for optimizing the location of *mega-sites* and allocating vaccines to the public. We consider a disease outbreak context in which a newly developed vaccine is introduced under conditions of limited supply. In the optimization problem, we focus on two questions: (1) where should the public health department set up *mega-sites* for large vaccine dispensing; and (2) how to assign individuals for vaccination. We assume that the *mega-site* locations will not change during the analysis period. We assume each *mega-site* has a maximum number of vaccines to dispense in each time epoch. We assume each region can have at most one *mega-site*. Regions can be defined in a variety of ways, and one convenient way may be using pre-existing definitions by health decision-makers; for instance, in LAC, ‘Health Districts’ are used to delineate health resources by geography [82]. We will therefore use the term Health District (HD) to represent regions within an area.

### Minimization of travel inconvenience considering commuting patterns

A model that does not account for commuting patterns may be imprecise in estimating the travel inconvenience. Therefore, in this section, we consider two categories of individuals in our population for vaccine assignments: commuters and non-commuters. In this work, a commuter is defined as a person who regularly travels some distance between their home and place of work every day. This allows a more precise estimation of cost quantification. In this study, we let $\mathcal {H}$ denote the set of all HDs. We use $e_{uv}$ to denote the number of commuters traveling from HD $u \in \mathcal {H}$ to HD $v \in \mathcal {H}$. The non-commuter population in each HD $u \in \mathcal {H}$ is defined as the total population, denoted by $p_u$, minus the commuters from HD *u*. We compute travel inconvenience for commuters based on distances from vaccine sites to both their residence and their workplaces. For instance, if an individual resides in HD *u* and commutes to their workplace in HD *v*, a vaccination site situated near either HD *u* or HD *v* should be regarded as convenient for that person.

The additional travel inconvenience for non-commuters is $c_{uw}+c_{wu}$ for people who live in HD $u\in \mathcal {H}$ and get vaccinated at HD $w\in \mathcal {H}$. For commuters, four different possible cases can be considered. An individual who lives in HD $u\in \mathcal {H}$, goes to work in HD $v\in \mathcal {H}$, and gets vaccinated at HD $w\in \mathcal {H}$ can choose to get vaccinated either before or after work, during work hours, en route to work, or while returning from work. We use $d_{uvw}$ to denote the additional travel inconvenience (minutes) for people who live in HD $u\in \mathcal {H}$, go to work in HD $v\in \mathcal {H}$, and get vaccinated at HD $w\in \mathcal {H}$. Therefore, the additional travel inconvenience for this individual is defined as $d_{uvw}=\min \{c_{uw}+c_{wu},c_{vw}+c_{wv}, c_{vw}+c_{wu}-c_{vu},c_{uw}+c_{wv}-c_{uv}\}$.

Our model distinguishes between commuting and non-commuting populations for two reasons. First, it allows for a more precise calculation of marginal travel costs: while non-commuter costs are a direct function of home-to-site distance, commuter costs are modeled as the minimum additional deviation from a pre-existing home-work trajectory. Second, this granularity ensures the model’s outputs are compatible with complex compartmental simulations, where transmission dynamics and contact patterns vary based on commuting status and associated behavioral factors.

We consider three decision variables for each time period within the set of all time periods $\mathcal {T}$. Let $x_u$ represent the decision on whether to open a *mega-site* in HD $u \in \mathcal {H}$. The variable $y_{uw}^t$ denotes the number of non-commuters residing in HD $u \in \mathcal {H}$ that are assigned to get vaccinated at HD $w \in \mathcal {H}$ during decision period $t \in \mathcal {T}$. Similarly, $z_{uvw}^t$ represents the number of commuters who live in HD $u \in \mathcal {H}$, work in HD $v \in \mathcal {H}$, and are assigned to get vaccinated at HD $w \in \mathcal {H}$ during decision period $t \in \mathcal {T}$.

Furthermore, the total number of *mega-sites* is restricted to not exceed *K*. Finally, let $U_{tw}$ denote the number of vaccines available at *mega-site* $w \in \mathcal {H}$ in decision period *t*. The detailed description of the notation is described in Table 1.

**Table 1:** Table of notation

	Notation	Definition
Set	$\mathcal {H}$	a set of HDs.
	$\mathcal {T}$	a set of decision periods.
Parameters	*K*	total number of *mega-sites* that can be opened.
	$c_{uv}$	the travel inconvenience (minutes) from HD $u\in \mathcal {H}$ to HD $v\in \mathcal {H}$.
	$d_{uvw}$	the additional travel inconvenience (minutes) for people who live in HD $u\in \mathcal {H}$, go to work in HD $v\in \mathcal {H}$, and get vaccinated at HD $w\in \mathcal {H}$.
	$p_u$	total population at HD $u\in \mathcal {H}$.
	$e_{uv}$	number of people who live in HD $u\in \mathcal {H}$ and go to work at HD $v\in \mathcal {H}$.
	$U_{tw}$	number of vaccines available at the *mega-site* $w\in \mathcal {H}$ for decision period $t \in \mathcal {T}$.
Variables	$x_u$	open a *mega-site* in HD $u\in \mathcal {H}$.
	$y_{uw}^t$	number of non-commuters who live in HD $u\in \mathcal {H}$ that are assigned to get vaccinated at HD $w \in \mathcal {H}$ during the decision period $t\in \mathcal {T}$.
	$z_{uvw}^t$	number of commuters who live in HD $u\in \mathcal {H}$, go to work in HD $v\in \mathcal {H}$ that are assigned to get vaccinated at HD $w \in \mathcal {H}$ during the decision period $t\in \mathcal {T}$.

We consider a traditional mixed integer linear programming (MILP) problem that minimizes additional travel inconvenience:

$$\begin{aligned}&\text {(P1)}\quad \min _{x,y,z} \quad \sum _{t\in \mathcal {T}}\sum _{u\in \mathcal {H}}\sum _{w\in \mathcal {H}}y_{uw}^t(c_{uw}+c_{wu})\\&\quad + \sum _{t\in \mathcal {T}}\sum _{u\in \mathcal {H}}\sum _{v\in \mathcal {H}}\sum _{w\in \mathcal {H}}z_{uvw}^td_{uvw} \end{aligned}$$

1a $$\begin{aligned} s.t. \quad&\sum _{u\in \mathcal {H}}x_u \le K\end{aligned}$$

1b $$\begin{aligned}&{\sum _{t \in \mathcal {T}} \sum _{w \in \mathcal {H}} y^t_{uw} + \sum _{v \in \mathcal {H}} \sum _{t \in \mathcal {T}} \sum _{w \in \mathcal {H}} z^t_{uvw} = p_u, \quad \forall u \in \mathcal {H}}\end{aligned}$$

1c $$\begin{aligned}&{\sum _{u\in \mathcal {H}}y_{uw}^t+\sum _{u\in \mathcal {H}}\sum _{v\in \mathcal {H}}z_{uvw}^t\le U_{tw}x_{w},\forall w\in \mathcal {H},t\in \mathcal {T}}\end{aligned}$$

1d $$\begin{aligned}&x_u \in \{0,1\}, y_{uw}^t\in \mathbb {Z}^+, z_{uvw}^t\in \mathbb {Z}^+,\forall u\in \mathcal {H},\nonumber \\&\quad v\in \mathcal {H},w\in \mathcal {H},t \in \mathcal {T} \end{aligned}$$

The objective in P1 is minimizing the total travel inconvenience from home/workplaces to vaccine sites. Constraint 1a limits the number of *mega-sites* that can be built. In this paper, we assume that all people eventually get vaccinated in one of the *mega-sites* during one of the decision periods $t \in \mathcal {T}$, and constraint 1b ensures that all individuals are vaccinated eventually. Constraint 1c ensures that individuals can only get vaccinated in an HD where a *mega-site* exists and restricts the number of vaccines that can be administered by the *mega-site* at the HD $w\in \mathcal {H}$ during the decision period $t\in \mathcal {T}$.

### Modeling disease dynamics

The MILP formulation in P1 only minimizes the total travel inconvenience. However, we are also interested in reducing poor health outcomes due to infectious disease in this population. In this section, we discuss how to integrate the public health objective into the optimization model using compartmental model and herd immunity analyses.

#### Compartmental model

Vaccines can prevent downstream transmission of disease, so it is important to track disease dynamics over time in order to understand the impact of *mega-site* location choices and allocation strategies. Compartmental models are commonly used for modeling complex disease dynamics of infectious disease on a population level [40–45]. Due to the complexity of disease dynamics, compartmental models are hard to formulate and often intractable except through simulation. We therefore use a simple susceptible-infectious-recovered (SIR) model with vaccinations which simplifies the disease dynamics to create a tractable objective term, we then use a more detailed compartmental model for evaluating the performance of our model. SIR models have been used extensively in many disease contexts and track health states, transmission, and recovery over time [83–85]. Our model is additionally stratified by HD to track the number of individuals within each HD in each health state.

In the model, we use $s^v,i^v,r^v$ to represent the proportion of individuals in each health state (susceptible (*s*), infectious (*i*), and recovered (*r*)) in HD $v\in \mathcal {H}$. We capture the transmission rate between HDs using $\beta _{uv},\forall u\in \mathcal {H},\forall v \in \mathcal {H}$, which denotes the transmission rate from HD *u* to HD *v*. We assume γ is the geographically invariant clearance rate. Our compartmental model is then:

2 $$\begin{aligned}&\frac{ds^{v}}{dt}=- \sum _u {\beta }_{uv} s^v (t) {{i}^u(t)}\nonumber \\&\frac{di^v}{dt} = \sum _u {\beta }_{uv} s^v (t){{i}^u(t)}-{\gamma i^v(t)}\\&\frac{dr^v}{dt} = \gamma i^v(t).\nonumber \end{aligned}$$

In Eq. 2, the first equation describes the rate at which the susceptible population in region *v* decreases. the second equation tracks the net change in the infected population within region *v*. The third equation represents the rate of recovery in region *v*.

#### Herd immunity threshold analysis

Much of the benefits of a population vaccination campaign can be garnered before all individuals have been vaccinated. In epidemiology, the *herd immunity threshold* is the idea that less than 100% of the population need be immune to the disease to prevent an epidemic [86]. We will use this concept to formulate our vaccine threshold targets.

Many works have studied computation methods for identifying herd immunity thresholds [86–88] within a population. We follow the method in [86] to compute the herd immunity threshold. We find the herd immunity threshold such that the spread of disease will start to decrease.

Then prevalence decreases as $\frac{di^v}{dt}<0, \forall v\in \mathcal {H}$. This is achieved when $s^v(t)<\frac{\gamma i^v(t)}{\sum _{u}\beta _{uv} i^u(t)},\forall v \in \mathcal {H}.$ The herd immunity threshold is the lowest herd immunity level required for the epidemic to decrease. Although herd immunity can be achieved through a rise in the number of recovered or via vaccinations [86], in our paper, we presume that herd immunity is primarily attained through vaccination, given that the number of infected and recovered individuals are relatively small compared to the entire population, particularly as we are interested in the initial stages of a pandemic. Let us define $L^v$ to be the herd immunity level that leads the epidemic to decrease. Then $L^v$ can be approximated by $1-s^v(t)$ at time *t* for region *v*. $L^v=1-s^v(t)>1-\frac{\gamma i^v(t)}{\sum _{u}\beta _{uv} i^u(t)},\forall v \in \mathcal {H}.$ We then simplify by assuming that the ratio of prevalences (denoted as κ) between HDs is a time-invariant constant, and this has been validated in the calibrated model (see Appendix A.2). Viboud et al. (2006) [89] show that synchronized influenza dynamics across regions. This provides additional support for this assumption. We use the average ratio over a specific period $[t^-, t^+]$ to represent this constant. Specifically, we define:

$$\begin{aligned} \kappa _{uv} \!=\! \frac{1}{\# \,\text {of time points in }[t^- ,t^+]} \sum _{t=t^-}^{t^+} \frac{i^u(t)}{i^v(t)}, \quad \forall u \in \mathcal {H}, \forall v \in \mathcal {H}, \end{aligned}$$

where time points within the interval $[t^-, t^+]$ are uniformly discretized. While the exact values may vary slightly with different discretization levels, the results should remain consistent overall, and higher-resolution discretization typically improves accuracy.

We therefore set the vaccination target for each HD *u* as the herd immunity threshold $L^{u*}=\frac{\sum _{v\in \mathcal {H}} \beta _{vu}\kappa _{vu}-\gamma }{\sum _{v\in \mathcal {H}} \beta _{vu}\kappa _{vu}}$ and incorporate these vaccination targets $L^{u*}$ into our optimization objective. We wish to minimize the difference between vaccination levels and $L^{u*}$ targets across all time:

$$\begin{aligned} p_u L^{u*}-\sum _{t'=1}^{t}(\sum _w y_{uw}^{t'}+\sum _v\sum _w z_{uvw}^{t'}), u\in \mathcal {H},t\in \mathcal {T}. \end{aligned}$$

We add this term to the optimization problem to make it a multi-objective formulation (see formulation P2 below).

This objective considers equal weights for each HD in the allocation process. However, we may want to prioritize HDs with residents at higher risk of becoming infected; in this work, we will consider transmission risk proxied by greater transportation movement. In general, weighting each HD by a risk score could capture a wide variety of definitions of risk (due to demographic characteristics, e.g., age, or differences in access to treatment, etc.). We term these weights ‘prioritization scores’ as they allow the optimization model to prioritize specific regions and allocate vaccinations to these areas preferentially. Such prioritization scores for areas with different risks have been used in prior literature to help capture heterogeneity in disease control [73, 90].

We use $\delta _u^t$ to define the prioritization score for HD $u\in \mathcal {H}$ at time period $t\in \mathcal {T}$. In Section 3, we formally define our prioritization score, evaluate its performance, and compare it with other commonly used scores in the literature.

#### Equitable distribution of vaccines

We also need to consider vaccination distribution equity. Access to healthcare resources without distinction between individuals is a fundamental tenet of promoting human rights [91, 92]. Thus, in our optimization model, we aim to reduce the disparity in the number of individuals vaccinated across various HDs during the initial vaccination phase ($\mathcal {T}_E\subset \mathcal {T}$) when supply is mostly available through *mega-sites* and not available in local clinics. As the proportion of vaccinated individuals grows and the urgency of vaccination wanes due to protective effects from herd immunity, equity may be a less pressing concern. Of course, $\mathcal {T}_E$ can be set equal to $\mathcal {T}$ if equity objectives should be considered throughout the time horizon. We adopt an equity measure that penalizes the gap between the most and least covered regions, following a similar approach to Bertsimas et al. (2021) [9] and Balcik et al. (2022) [51].

### A multi-objective model

Realizing the need to prioritize high risk groups of becoming ill, promote equitable distribution of vaccines, as well as improve accessibility to vaccination, we formulate another model that incorporates these objectives. Let $\zeta ^t_u$ and $\tau _t$ present the objective value on public health outcome at time *t* for HD *u* and equitable distribution at time *t* respectively, we have a MILP formulation as follows:

$$\begin{aligned}&\text {(P2)} \quad \min _{x,y,z} \quad \sum _{t\in \mathcal {T}}\sum _{u\in \mathcal {H}}\sum _{w\in \mathcal {H}}y_{uw}^t(c_{uw}+c_{wu})\\&\quad + \sum _{t\in \mathcal {T}}\sum _{u\in \mathcal {H}}\sum _{v\in \mathcal {H}}\sum _{w\in \mathcal {H}}z_{uvw}^td_{uvw}+\lambda \sum _{t\in \mathcal {T}} \sum _{u\in \mathcal {H}} \delta ^t_u \zeta _u^t+\lambda ' \sum _{t\in \mathcal {T}_E}\tau _t \end{aligned}$$

2a $$\begin{aligned} s.t. \quad&\text {Constraints in P}1\end{aligned}$$

2b $$\begin{aligned}&\zeta _u^t\ge p_u L^{u*}-\sum _{t'=1}^t(\sum _{w\in \mathcal {H}} y_{uw}^{t'}\nonumber \\&\quad +\sum _{v\in \mathcal {H}}\sum _{w\in \mathcal {H}} z_{uvw}^{t'}),\forall u\in \mathcal {H},t\in \mathcal {T}\end{aligned}$$

2c $$\begin{aligned}&\tau _t \ge \frac{(\sum _{v\in \mathcal {H}}\sum _{w\in \mathcal {H}} z_{uvw}^t+\sum _{v\in \mathcal {H}} y_{uv}^t)}{p_u}\nonumber \\&\quad -\frac{(\sum _{v\in \mathcal {H}}\sum _{w\in \mathcal {H}} z_{u'vw}^t+\sum _{v\in \mathcal {H}} y_{u'v}^t)}{p_{u'}},\nonumber \\&\quad \forall u\in \mathcal {H},u'\in \mathcal {H},t\in \mathcal {T}_E\end{aligned}$$

2d $$\begin{aligned}&\zeta _u^t\ge 0,\forall u \in \mathcal {H},t\in \mathcal {T} \end{aligned}$$

The notation used in P2, in addition to Table 1, is listed in Table 3. Unlike P1 in Section 2.1, this new MILP model (P2) includes a public health objective as well as an equitable distribution objective. In the objective, $$\zeta _u^t$$ is the remaining unvaccinated population at HD $$u\in \mathcal {H}$$ at time $$t\in \mathcal {T}$$ relative to the vaccination threshold target $$L^{u*}$$. $$\tau _t$$ denotes the maximum difference in the proportion of vaccines distributed between any two HDs at time period *t*, during the early vaccination phase $$\mathcal {T}_E \subset \mathcal {T}$$. This is analogous to the concept of statistical parity, which inherently relies on a relative measure to evaluate proportional fairness across groups. Therefore, unlike constraint 2b, constraint 2c is formulated using a relative measure. $\lambda^{\prime }$ balances the importance of equitable distribution and prioritization over groups with higher risks of becoming infectious. λ balances the importance of travel inconvenience and public health outcomes. Constraints 2b and 2c compute the value of $$\zeta _u^t,\tau _t$$ from the vaccine allocation strategy.

To formulate Problem P2, we require a calibrated SIR model to compute herd immunity thresholds, as well as estimates of the number of commuters and non-commuters, their associated cost matrices within the target region, the number of planned mega-sites, and the capacity of each site. We describe how these parameters can be derived from real-world data in Section 3. As this is a multi-objective optimization model, two hyperparameters, λ and $\lambda^{\prime }$, must be specified. Different values for these hyperparameters yield different solutions due to their influence on the relative weighting of the objective components. These hyperparameters depend on the magnitude and context of the specific problem instance, which are not scale-free. We discuss how to choose appropriate ranges for these hyperparameters in practice in Section 3.

### Evaluating public health outcomes

We have incorporated public health outcomes in the optimization problem objective using an SIR model, so we should evaluate the effectiveness of our vaccine allocation strategy in a more realistic way. We therefore use a more detailed compartmental model that more closely captures the empirically observed disease trends, as a simpler model could lead to inaccuracies in capturing the dynamics [93]. We formulate a compartmental model that includes the following health and treatment states as shown in Fig. 3: susceptible (S), vaccinated (V), exposed (E), identified infectious (I), unidentified infectious (U), hospitalized (H), recovered (R), and dead (D). Each of these states is stratified by HDs to track variation in disease outcomes by region. We assume full immunity will be achieved after the first dose of vaccination. We assume that only transmission rates (β) and vaccination rates (ξ) vary geographically by HD. This compartmental model can also be represented as a system of ordinary differential equations, which is shown in Appendix A.1.

We use cumulative cases (*C*), cases (*I*), cumulative deaths (*D*), and hospitalizations (*H*) [94] to calibrate the compartmental model. We hereafter use ‘ $\hat{phantom{0}}$ ’ to represent simulated outcomes from the compartmental model to clearly differentiate them from empirical surveillance report values. The calibration objective is to minimize the error in the simulated cumulative cases ($\hat{D}+\hat{H}+\hat{R}+\hat{I}$), cases ($\hat{I}$), cumulative deaths ($\hat{D}$), and hospitalizations ($\hat{H}$).

We first calibrated the model without vaccinations. The calibrated parameters $\textbf{p}$ include all rate parameters from one compartment to the other. These parameters are calibrated using Least absolute deviations (LAD) on the following objective:

$$\begin{aligned} \min _{\textbf{p}}&\ \ w_D\sum _{u\in \mathcal {H}} \sum _{t} \bar{w}_u(|\hat{D}_{u,t} \!-\! D_{u,t}|)+w_C\sum _{u\in \mathcal {H}} \sum _{t} \bar{w}_u(|\hat{D}_{u,t}\\&\quad +\hat{H}_{u,t}+\hat{R}_{u,t}+\hat{I}_{u,t} - C_{u,t}|)\\&\ \ + w_I\sum _{u\in \mathcal {H}}\sum _{t} \bar{w}_u(|\hat{I}_{u,t}- I_{u,t}|)\\&\quad +w_H\sum _{u\in \mathcal {H}} \sum _{t} \bar{w}_u(|\hat{H}_{u,t}- H_{u,t}|)\\ s.t.&\ \ \text {Compartmental Model System Dynamics}\\&\quad \text {in Appendix Section}~A.1 \end{aligned}$$

In the LAD, four calibration targets are minimized: cumulative death, cumulative identified infections, identified infections over time, and hospitalizations, where all calibration targets are at HD level. $w_D,w_C,w_I,w_H$ are weights for different calibration objectives (Detailed values are provided in Appendix A.2). The weight $\bar{w}_u$ for each HD *u* is determined by comparing its total population to the total population in LAC. $\hat{H}_{u,t}, \hat{D}_{u,t}, \hat{R}_{u,t}, \hat{I}_{u,t}$ are the number of hospitalizations, deaths, recovered, and identified infections from the compartmental model for HD $u\in \mathcal {H}$ at time *t*. $D_{u,t}, C_{u,t}$ (cumulative identified cases for HD *u* at time *t*), $I_{u,t}, H_{u,t}$ are the actual number of deaths, cumulative identified infections, identified infections, and hospitalizations for HD $u\in \mathcal {H}$ at time *t*.

We use this calibrated compartmental model to compute the number of infections averted under different *mega-site* placement and vaccine allocation scenarios (shown in Algorithm 1). To do this, we first compute the number of cumulative infections with no vaccinations ($N_{CI}$). Then we compute the number of cumulative infections from the compartmental model with a given vaccination strategy π ($\bar{N}_{CI}^{\pi }$). To evaluate the vaccination program π’s efficiency in reducing the disease burden, we then calculate the number of infections averted by $N_{CI}-\bar{N}_{CI}^{\pi }$.

**Algorithm 1:**
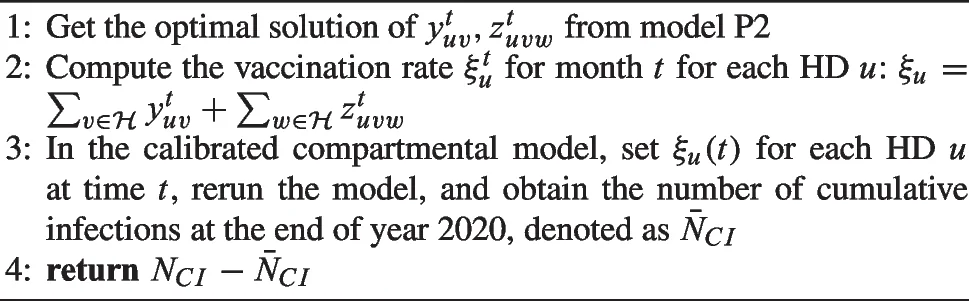
Computing Infections Averted.

## Numerical example

When vaccines became available several months after the COVID-19 outbreak, Los Angeles County (LAC) initiated a vaccination program which used large venues such as parking lots, universities, and theme parks, referred to as ‘mega-sites,’ to facilitate mass vaccination efforts [95]. LAC used a total of six *mega-sites* between January to April, 2021. LAC had a sign-up system where residents provided demographic information and signed up for available *mega-site* appointments [96]; availability at different sites and times could be restricted depending on demographic characteristics such as age and work industry. This system essentially assigned residents to mega-sites according to rules which changed over time as vaccination restrictions were lifted to prioritize vulnerable residents [97]. In this period, each *mega-site* was able to administer between 200,000 to 800,000 vaccinations per month.

Using the disease modeling framework and optimization models presented in Section 2, we outline the model’s parameterization, evaluate the model’s performance by the number of infections averted, travel inconvenience, and equitable distribution of vaccines among the 26 HDs within LAC, and compare results to those using the empirically chosen locations in 2021.

### Model inputs

We use traffic data from Performance Measurement System (PeMS) Data Source [98], Open Route API [99], and population data [100, 101] to parameterize the optimization model. We assume six *mega-sites* can be built at maximum. We assume each can provide a capacity of 400,000 monthly vaccinations. The optimization model solves monthly decisions across a six months time horizon. In the rest of Section 3.1, we provide a comprehensive explanation of model inputs.

#### Commuters and non-commuters

We use traffic volume data from PeMS database [98] to compute the number of daily commuters from one HD to another. PeMS contains the number of vehicles passing over a sensor in 5 minutes intervals and the average speed of these vehicles. We approximate the number of vehicles traveling from one region to another region by extending a previously published dynamic origin-destination estimation (DODE) model [102, 103]. A heatmap of the estimated traffic flow of 2,876,523 vehicles on a daily basis is given in Appendix B.2. In 2020, there were an estimated 4,396,232 commuters across LAC [101]. Given evidence suggesting a dramatic decrease in public transportation usage during that year [104], we assume that people used cars to commute rather than public transportation. We also assume that people did not walk to mega-sites for vaccination; indeed, LAC offered drive-through-only vaccinations at these empirical mega-sites. Therefore, to obtain a matrix representing the number of commuters in LAC, we scale up the vehicle matrix by multiplying all entries by 1.53–the average number of people per vehicle. The number of commuters in each HD can then be computed by summing the corresponding row of the commuter matrix. Then the number of non-commuters can be obtained by taking the difference from the total population in each HD [100].

#### Parameterization of travel cost matrix

For an individual who lives in or works in a certain HD, we use the geographic centroid of that HD to represent the home or work address. Similarly, we use the geographic centroid of each HD to approximate the potential location of the *mega-site* in an HD. To parameterize the cost of travel from one HD $u\in \mathcal {H}$ to another HD $v\in \mathcal {H}$, we use Open Route API [99] to compute the time (in minutes) needed to traveling from *u* to *v* ($c_{uv}$) under traffic-free conditions. We use these values to parameterize the travel cost matrices (*c*, *d*) for commuters and non-commuters as described in Section 2.1.

#### Herd immunity threshold

We calibrated a compartmental model to estimate the transmission and recovery rates. Using these calibrated rates and the number of infectious individuals from the model, we then calculated the herd immunity threshold. When computing the ratio of prevalence $\kappa _{uv}$ between HD *u* and *v*, we use time periods of one day. We find the vaccination target for each HD to ensure herd immunity using $L^{u*},\forall u \in \mathcal {H}$ in Section 2.2.2. High vaccination targets are observed for all HDs due to the relatively high transmission rate compared to the recovery rate. Among all HDs, West Valley has the lowest vaccination target (95.13%), and Pasadena has the highest vaccination target (99.15%). On average, 97.36% of the total population needs to be vaccinated to achieve herd immunity. Detailed herd immunity thresholds across HDs are shown in Appendix B.3. However, the herd immunity threshold does not dictate prioritization within the population when no health districts (HDs) have reached the threshold or when all HDs have met the target. We therefore introduce the prioritization score in the next section.

#### Prioritization score

In formulation P2, we weight the herd immunity level needed in each HD by a prioritization score ($\delta _u^t$) to preferentially target more vulnerable HDs first. Prior work has demonstrated that geographically targeted prioritization vaccination strategies for vaccine allocation to groups at high risk of becoming infected can be effective at reducing disease burden [70, 71]. Measuring social interaction levels can be one measure to identify groups at a higher risk of infection [72]. Similar to Hu et al. (2021) [105], we prioritize vaccination accessibility for regions that have greater traffic flow. Specifically, we let the score be the number of daily commuters in each HD divided by the total number of commuters in LAC, $\delta _u^t = c(t) \frac{\sum _{v\in \mathcal {H}}e_{uv}}{\sum _{u,v\in \mathcal {H}}e_{uv}},$ where *c*(*t*) is the time-dependent discount factor. We apply a discount factor to the prioritization score to reduce the value of future vaccination rewards compared to immediate vaccination. In our experiment, we chose to use traffic intensities as opposed to calibrated transmission rates as they offer greater flexibility—for example, we can use the prioritization scores based on recent weekly or monthly traffic data without needing to recalibrate the entire model.

In the Appendix C.2, we compare our prioritization score with others from the literature. We found that the score based on commuting patterns resulted in significantly more infections averted than the others, while its performance on the two other metrics was similar. Therefore, we use traffic flow to define the prioritization score.

To sum up, the determination of *mega-sites* requires knowing traffic flow, commuter and non-commuter population sizes, and herd immunity thresholds. Traffic flow is commonly estimated using transportation databases, population statistics are usually obtained from census databases, and herd immunity thresholds are calculated based on data from disease surveillance systems.

**Fig. 1:**
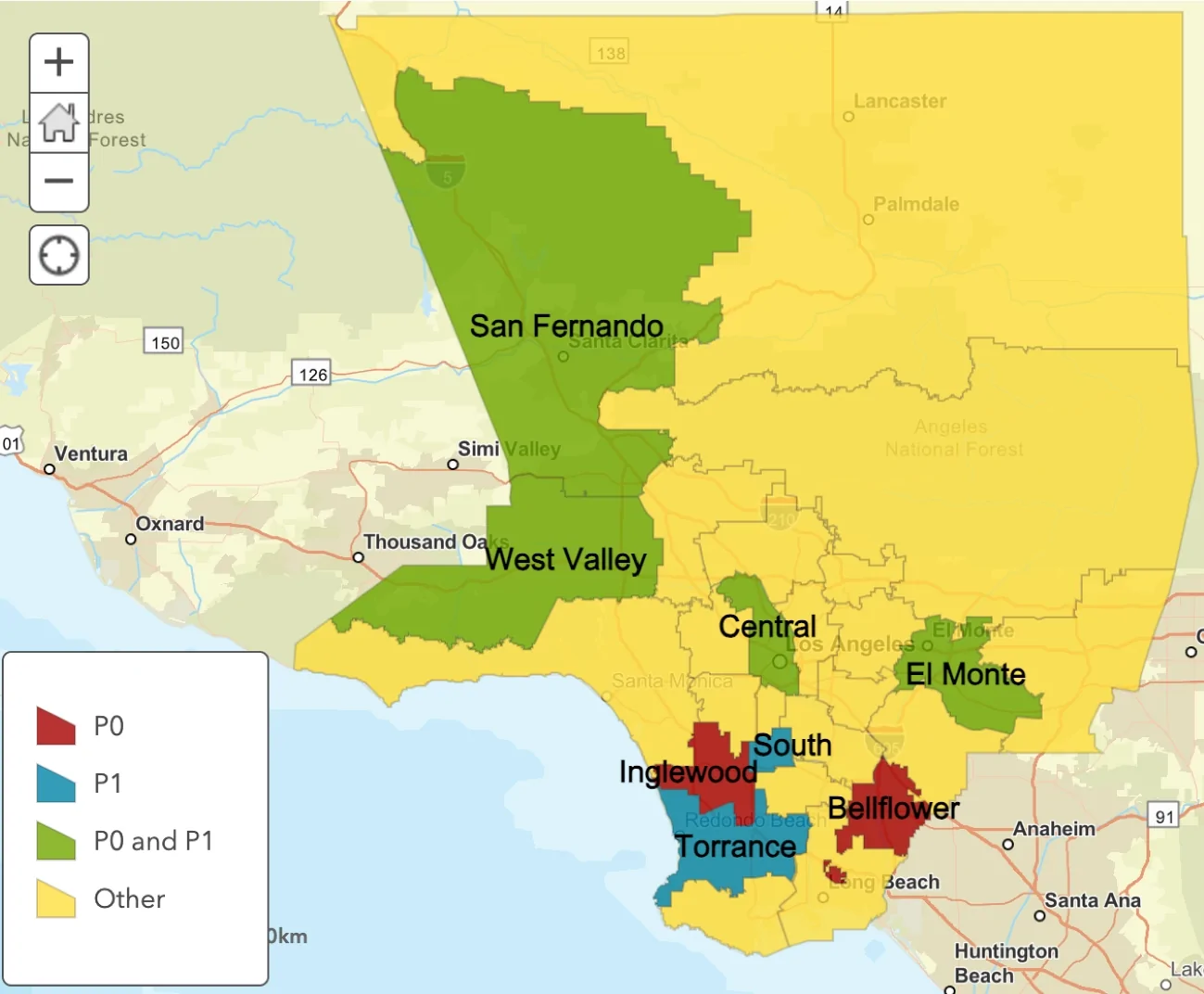
A map of 26 health districts in LAC, with HDs with *mega-sites* selected by P0 marked in red and green, HDs with *mega-sites* selected by P1 marked in blue and green, and HDs without *mega-sites* marked in yellow

### Calibrating compartmental model in LAC

We use publicly available COVID-19 cumulative cases (*C*), cases (*I*), cumulative deaths (*D*), and hospitalizations (*H*) [94] to calibrate the compartmental model. We consider the simulation to start on March 1st, 2020 with a 300-day calibration period. Within the calibration interval, we define five intervals in which calibration parameters are allowed to change. These five intervals are Lock-down (45 days), social events (45 days), re-opening (45 days), second wave (75 days), and holiday season (90 days). We calibrate the model sequentially on each of the calibration intervals listed above. The calibrated parameter set from the previous interval is used as the initialization parameter set for the next calibration interval. The calibration results are shown in Appendix A.2. Although we employed a simplified model to calculate the herd immunity threshold, we empirically verified that this threshold still holds when applied to our more complex compartmental model.

To compute the number of infections averted, we first compute the number of cumulative infections with no vaccinations ($N_{CI}=625,737$). Then we compute the number of cumulative infections by the end, assuming that the vaccination starts 6 months prior to the end — during the reopen interval.

### Model results

Traditional facility location models [13, 15–17] generally do not capture commuting patterns in *mega-sites* selection. To highlight the impact of incorporating such patterns, we first consider a baseline transportation model that assumes all individuals are non-commuters (i.e., $$e_{uv} = 0$$ for all $$u, v \in \mathcal {H}$$), referred to as P0. We begin by solving P0 and then compare it to our proposed model, which incorporates commuting patterns into the estimation of travel inconvenience (P1). We then solve a model with both travel inconvenience and public health outcome objectives (P2) to understand how health and equity objectives change our solution. We solve all models using Gurobi 9.5.2 on Python 3.9. We assume the importance weight on the public health objective (λ) is 9, the weight on the smoothness objective ($\lambda^{\prime }$) is 150 multiplied by the average population across HDs, and the discount factor (*c*(*t*)) is $$0.9^t$$. These parameters will be varied in sensitivity analyses. We assume the capacity for each *mega-site* is uniformly 400,000 vaccinations per decision period, and each decision period spans a month, with a total of six such periods. The choices for the number of vaccination centers and their capacities are similar to the empirical setup [95–97]. We also compared the integer solution with its continuous relaxation of the *y* and *z* variables and found the selection of *mega-sits* is the same, with less than 0.01% of the assignments being changed. However, 87 fewer individuals received vaccination due to rounding. Because the integer model solves within 50 minutes and computational efficiency is not our primary focus, these comparisons are omitted from the main text. Interested readers can find more details in the Appendix C.4.

**Fig. 2:**
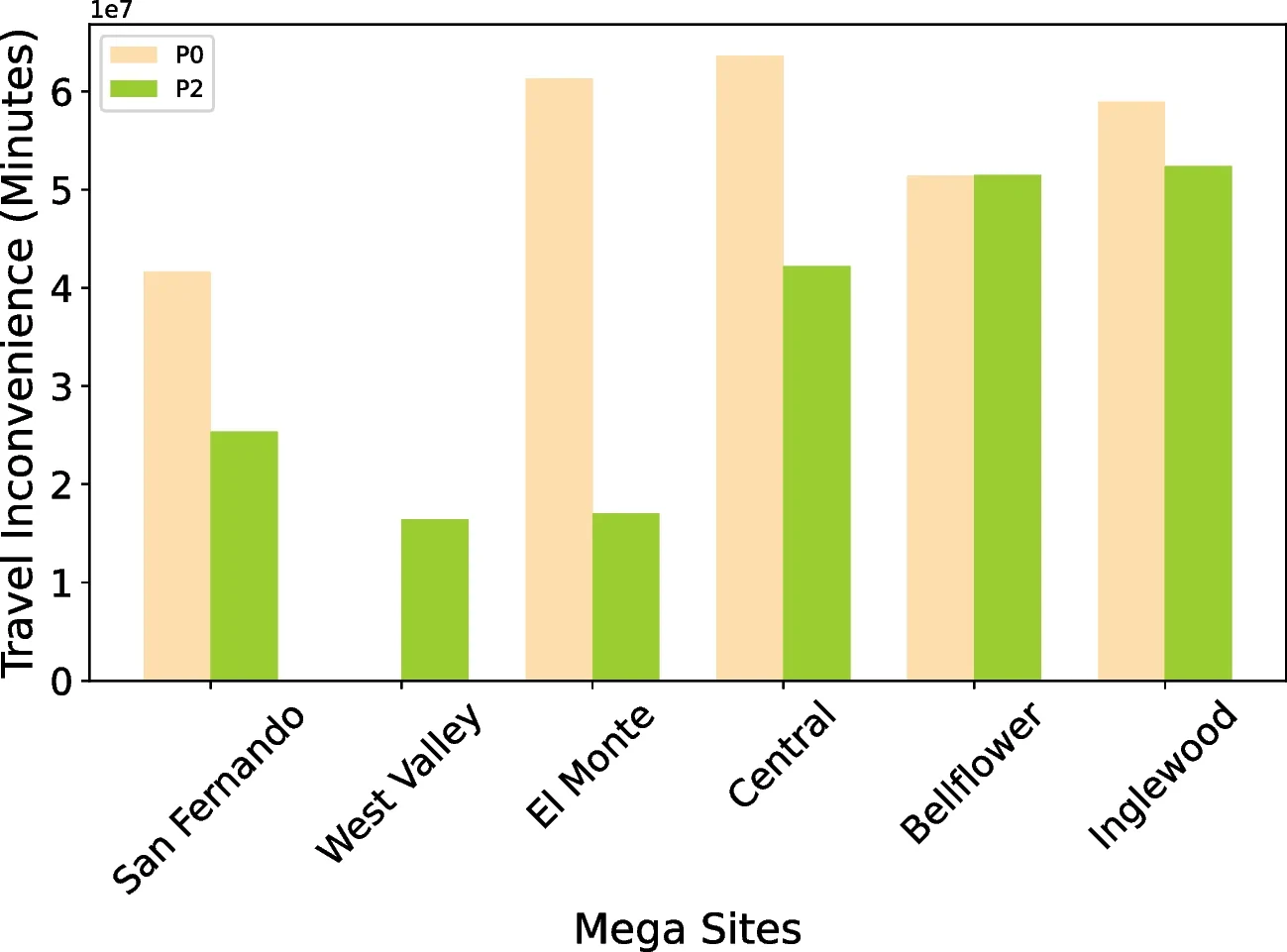
Total travel inconvenience by each *mega-sites*. It is zero for individuals assigned to West Valley for P0 as it assigns only internal individuals

**Table 2:** Model results

Model	Travel inconvenience (mins.)	Infections averted	Inequity objective
P0	276,949,555	374,538	2
P1	196,572,213	390,252	1.603
P2	204,859,537	408,084	0.831

#### Effects of integrating commuting patterns

In this section, we study how integrating commuting patterns changes the solution of *mega-sites* and vaccine allocation, and how these changes impact travel inconvenience and public health outcomes.

In the traditional model that minimizes the travel inconvenience only from homes (P0), San Fernando, West Valley, Central, El Monte, Inglewood, and Bellflower (shown in Fig. 1) are selected as locations of *mega-sites*. The model incorporating commuting patterns (P1) selects four of the same locations as P0 (see Fig. 1). The two differences in location (Torrance and South) result in locations that are generally closer to major freeways in LAC (both Torrance and South are closer to I110 and I405 than Bellflower). P1 locations should therefore be more convenient for commuters, evidenced by the substantially reduced total travel inconvenience (nearly 30% reduction from 276,949,555 minutes to 196,572,213 minutes).

To evaluate and compare public health outcomes between P0 and P1, we find the number of monthly vaccinations in each HD from the optimization model. We use this value to calculate the HD-specific vaccination rate in the compartmental model, as described in Section 2.2.1. Finally, we ran the comprehensive compartmental model to identify the infections averted at the end of 2020 using these vaccination rates (compared to the counterfactual where no vaccination was offered). In the P0 model, 374,538 infections were averted by the end of the year 2020 while the P1 model averted 390,252 infections (4% more than in the P0 model). This indicates that incorporating commuting patterns in this example also improves disease control even if public health outcomes are not in the objective formulation.

The inequity objective measures the sum of the maximal difference between HDs with the largest and the smallest vaccinated population percentage during $t\in \mathcal {T}_E$. In this study, we choose $\mathcal {T}_E$ to be the first two months of vaccination due to the high volume of vaccinations observed during the first two months in LAC [106]. Without incorporating inequity into the objective function, we observe large differences in vaccinations across HDs in the first two months. For instance, in the P0 solution, all individuals living in Central are vaccinated in the first month, while none of the individuals living in Pomona are vaccinated. In the second month, there are still zero vaccinations in Pomona, resulting in an inequity score of 2. P1 has a lower inequity score of 1.603 (20% less than in the P0 model).

#### Multi-objective model results

In this section, we present results of the optimization formulation (P2) that integrates objectives in P1 plus public health and equity objectives. We then compare these results with the previously discussed models.

P2 has the same recommendation as P0 on the locations of *mega-sites*, but the vaccine assignments between the two models are distinct. As shown in Fig. 2, the total travel inconvenience in minutes is smaller in P2 for all *mega-sites* except for those receiving vaccination in West Valley. El Monte experiences the most significant decrease, with a reduction of roughly 70 million minutes. In contrast, West Valley faces an increase in travel inconvenience. This is because P0 only assigns individuals who live in West Valley to the *mega-site* in West Valley, which results in zero total travel inconvenience for individuals vaccinated there. On the other hand, P1 assigns individuals who do not live or work near West Valley to West Valley as their more convenient locations are already at capacity, which results in higher total travel time. This increase is offset by decreases in travel inconvenience in other *mega-sites*.

Table 2 shows that formulation P2 is able to achieve improvements in infections averted and lower inequity while still achieving less travel inconvenience than formulation P0 and only a 4% increase from formulation P1. The P2 locations are able to prevent approximately 18,000 (6%) more infections than P1 by the end of year 2020 and achieve a 48% reduction in inequity compared to the P1 solution.

Additionally, we tested the model using weekly decision intervals and found that the selected *mega-sites* remained unchanged from the solution of P2, suggesting that the solution of P2 is not sensitive to a more frequent decision interval within a given range.

Moreover, we conducted a sensitivity analysis assuming immunity is achieved one month after vaccination (Tables 3, 4 and 5). We found that the selection of *mega-sites* remains unchanged. Additionally, we considered a scenario where vaccinated individuals have only an average protection of 80%, reflecting the reality that not all vaccines are 100% effective. We found that the selection of *mega-sites* remained consistent. These sensitivity analyses suggest that the locations recommended by the model are not sensitive to variations in the vaccine immunity threshold within a given range. However, the assignment of individuals to vaccination sites may vary, and the values of the three objectives also change accordingly (see Appendix C.3).

#### Benchmark solution: Empirical locations in LAC

To benchmark our model against a more realistic setup, we compare model outcomes to those if we fixed the *mega-site* locations to those actually used in LAC from January to May of 2021. More specifically, we solve the optimization problem P2 while fixing the $x_u$ variables to be these empirical *mega-sites* locations.

In the empirical location model, the *mega-sites* locations are San Fernando, Central, Northeast, Southeast, Inglewood, and San Antonio. Except for one *mega-site* in San Fernando, all other *mega-sites* are located around the Central district. Compared with the empirical *mega-sites* locations, P2 recommends a more dispersed distribution of *mega-sites*, with two *mega-sites* in the northwest, two around the Central district, and two in the east.

The empirical model incurs a 40% increase in total travel time compared to the P2 solution. Three point one percent (approximately 310,000) fewer individuals from the empirical solution can go to the most convenient *mega-sites* compared to P2. In the empirical model, 407,855 infections were averted by the end of the year 2020, approximately 200 less than the P2 solution. However, it has an inequity objective value of 0.721, which is less than that in P2. This indicates there is a trade-off between infections averted and equitable distribution.

Comparing both the results from the public health perspective and transportation perspective, our P2 formulation improves upon the single objective model P1 as well as the model with empirical locations, indicating that both accessibility and public health outcomes can be improved from the empirical setup.

### Sensitivity analyses

Recall the public health objective in our optimization model is minimizing $\lambda \sum _{t\in \mathcal {T}} \sum _{u\in \mathcal {H}} \delta ^t_u \zeta _u^t+\lambda '\sum _{t\in \mathcal {T}_E} \tau _t$, which considers prioritization in vaccination allocation ($\delta ^t_u$) and equity in distribution for the early phase $\mathcal {T}_E$. In this section, we vary the importance weight on equitable distribution and importance weight on public health objectives to understand how the allocation changes with respect to changes in objectives. We made several assumptions when designing our health objective term, so we tested whether our health objective was reasonable by assessing its correlation with with the number of infections averted.

#### Testing model assumptions: Evaluating the health objective term for concordance with cases averted

Our public health objective consists of vaccination targets (from a simplified SIR model) and the prioritization score (from commuting patterns in LAC). This objective serves as a proxy for true health goals such as infections averted. We evaluate the validity of this approximation by varying the importance of the health objective term (λ) and testing if the number of infections reduced is positively correlated to λ. A good health objective would avert more infections with increasing emphasis on the health objective. This can also serve as a sensitivity analysis.

When $\lambda \le 9$, the number of infections averted increases with λ. This suggests that the public health objective we have adopted serves as an effective proxy for the real-world goal of reducing infections. However, when λ surpasses 9, the number of infections averted plateaus and even slightly drops, implying that beyond a certain point of weight on the public health objective, the public health objective does not approximate the reduction in infections well. In those cases, when λ is exceedingly high, the optimization model prioritized the vaccines only for areas with high risks of becoming infectious while other areas with no vaccine access remain vulnerable, thus not reducing infections effectively.

#### Changes in importance weight on equitable distribution

We use the hyperparameter $\lambda^{\prime }$ (importance on equitable distribution) to balance between prioritization and equitable distribution. To understand how the importance of equitable distribution influences the solution, we varied $\lambda^{\prime }$ from 0 to 1000 multiplied by the average population (to match the magnitude of other objectives) across HDs while other hyperparameters remained the same.

Locations of *mega-sites* do not change when varying $\lambda^{\prime }$ in the P2 model. However, the inequity score drops from 1.96 to 0 as $\lambda^{\prime }$ increases from 0 to 250 multiplied by the average population across HDs. By more strongly weighting equitable distribution, the optimization model is configured to assign individuals for vaccination more uniformly across regions within each period.

The number of infections averted is 403,588 when $\lambda^{\prime }$ is 0, and 403,718 when $\lambda^{\prime }$ is 1000 multiplied by the average population across HDs, but is higher when $\lambda^{\prime }$ is 150 multiplied by the average population across HDs (408,084 infections averted). When not considering equitable distribution at all (i.e., $\lambda^{\prime }=0$), the optimization model prioritized vaccines only to areas at high risk of infection (higher transportation activities) while low-risk areas became more vulnerable due to limited access to the vaccination. The inequity objective value is 1.96 in this case, meaning that the sum of the maximal difference between HDs with the largest and the smallest vaccinated population percentage during the first two months is over 196%. This indicates a significant inequity in vaccine allocation across HDs. When the importance of equitable distribution overrides the other objectives, the optimization assigns individuals more uniformly for vaccination, which leads to an ineffective way of distributing resources and low number of infections averted.

### Generalization to future scenarios

To ensure objective magnitudes remain comparable across diverse applications, the penalty parameter $\lambda^{\prime }$ should be determined via a grid search over the range $$[0, 1000 \times \text {average population}]$$, while λ is ideally selected within the interval of zero and average commute time.

#### Number of *Mega-Sites*

We conducted an additional analysis on the number of sites to evaluate its impact on the model and to provide insights for the model in future contexts. We choose the number of *mega-sites* to six to benchmark with empirical solutions. We vary this number by considering five and seven *mega-sites*. In all cases, the entire population is eventually fully vaccinated. Reducing the number of *mega-sites* significantly decreases the infections averted. Increasing the number of *mega-sites* improves travel inconvenience and inequity. However, it does not lead to a notable improvement in the number of infections averted. These results suggest that the optimal site count is a function of vaccine supply, period-specific capacity, and total population. Given that LAC represents a high-density metropolitan baseline, our findings indicate that six *mega-sites* serve as an effective benchmark for regions of similar scale and density in future public health crises.

### The influence of travel inconvenience on vaccine acceptance

Prior work indicates that barriers to access vaccination sites, reflected in higher travel inconvenience, may deter individuals from becoming vaccinated [6, 7]. We may therefore be undervaluing the true benefit of reducing travel inconvenience in our models. To remedy this, in this section, we incorporated a likelihood function that computes an individual’s probability of choosing to get vaccinated based on their travel inconvenience. We re-evaluate the number of infections averted using the compartmental model after accounting for this additional increase in seeking vaccination. Specifically, in the compartmental model, we compute the vaccination rate (ξ) for each month based on the results from the optimization model. Now, if we take into account the willingness to vaccinate as a function of distance, we first calculate the likelihood ($l_{uvw}$) that an individual will choose to get vaccinated, given that they live in HD *u*, work in HD *v*, and are assigned to get vaccinated in HD *w*. Subsequently, the vaccination rate for individuals who live in *u*, work in *v*, and get vaccinated in *w* is adjusted to $\xi _{uvw} l_{uvw}$.

Following [107, 108], we model vaccination likelihood as a function of travel inconvenience. Let $d_{uvw}$ represent the minimum travel cost for an individual living in *u*, working in *v*, and vaccinated at site *w*, where $u,v,w \in \mathcal {H}$. We define a normalized relative convenience score, $\nu _{uvw}= \frac{\sum _{w'\in \mathcal {H}}(d_{uvw'}-d_{uvw})}{\max _{w'}{d_{uvw'}-\min _{w'}{d_{uvw'}}}}$. Then the probability $l_{uvw}$ of an individual choosing vaccination at site *w* is calculated via the logit function: $l_{uvw}=\frac{\exp (\nu _{uvw})}{1+\exp (\nu _{uvw})}$. These probabilities scale the HD-specific vaccination rates within the compartmental model to estimate cases averted.

Given that the decrease in travel inconvenience leads to more people choosing to get vaccinated, 3,813 cases would be averted due to greater access to vaccination using model P2 instead of the empirical setup, which now averts 399,016 infections. This is greater than the unadjusted benefit (an additional 229 cases averted when comparing the unadjusted P2 with the unadjusted empirical solution). Because the empirical solution substantially increases travel inconvenience compared to the P2 solution, fewer individuals tend to come to *mega-sites* for vaccination and thus leads to a worse public health outcome.

## Conclusion

We present an optimization formulation for selecting locations of vaccine sites. In the optimization model, we integrate multiple objectives including travel inconvenience, prioritization, and equitable distribution.

Our numeric case study on COVID-19 vaccination in LAC demonstrates that our model significantly eases travel burden and enhances public health outcomes compared to the empirical approach. It cuts down the total transportation time for vaccinations by roughly 26% for LAC residents. Furthermore, we contrast the empirical solution’s simulation results on public health outcomes with those from our optimized strategy; our model shows a reduction in infections by an additional 229 cases. When accounting for the influence of travel inconvenience on vaccine acceptance, our model demon-strates a reduction of an additional 3,813 infection cases.

The policy implications for our work are (1) locations of *mega-sites* for dispensing a large number of vaccinations are recommended to be placed widely across the region for reducing the overall travel inconvenience, and (2) merely minimizing travel inconvenience in determining vaccine site locations and vaccine distribution falls short – it’s also essential to identify and prioritize high infection risk areas to more effectively control disease transmission (3) balancing travel inconvenience, the number of infections prevented, and equity is challenging. However, with an appropriate setup in the optimization model, it’s possible to achieve satisfactory outcomes in all these aspects.

We must acknowledge several limitations of this work. While in reality, vaccines were not available until later, using July 2020 in our example allows us to study what would occur without the changing dynamics due to the real-world implementation of vaccine uptake (we can calibrate the model to non-vaccine data) but is close enough to the actual vaccine available date that we can compare between our optimal policy and real-world policies. We assume the capacities of different vaccination sites are uniform. We only consider individuals’ residence/work HD in vaccine assignment.

Our model assumes that all individuals receive their vaccinations at *mega-sites*. In practical terms, these *mega-sites* are operational primarily in the initial stages and are eventually phased out as vaccinations become available at local clinics. Our model assumes that individuals follow their assigned vaccination sites; however, in practice, individuals may prefer to visit the closest site. While travel time within larger HDs like San Fernando was not initially considered, a sensitivity analysis incorporating these factors demonstrated that the solution remained consistent. We acknowledge that the herd immunity level is subject to uncertainty due to assumptions made and uncertainty in disease dynamics. However, additional analyses using different herd immunity thresholds found that the selection of *mega-site* locations remained stable. Additionally, herd immunity thresholds tend to be similar across various diseases; for example, in our COVID-19 case study, we calculated an average herd immunity threshold close to 97%, similar to that of measles [109].

Despite these limitations, we believe that this work provides interesting insights into not only the locations of vaccine sites but also the allocation of vaccines. Our paper provides a tractably solvable optimization formulation and a numerical example to show how to reduce the travel burden and prevent more infections from the empirical approach. These results provide insight into future work on improving vaccination strategies to manage the disease burden in scenarios where an outbreak is ongoing and a new vaccine has just been introduced.

## Supplementary Information

Below is the link to the electronic supplementary material.

Supplementary file 1 (zip 6.49 MB)

## Figures and Tables

**Fig. 3:**
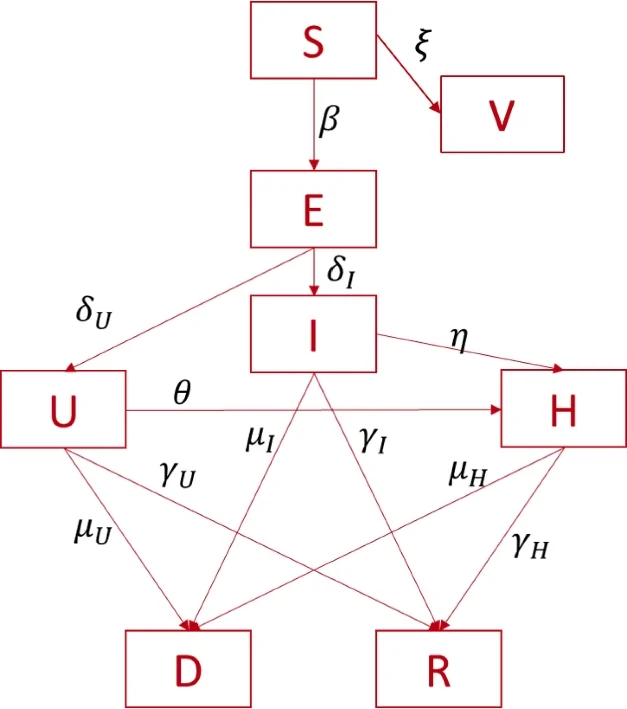
The compartmental model for modeling COVID-19 disease dynamics. Greek letters represent rates of flow between states over time

**Fig. 4:**
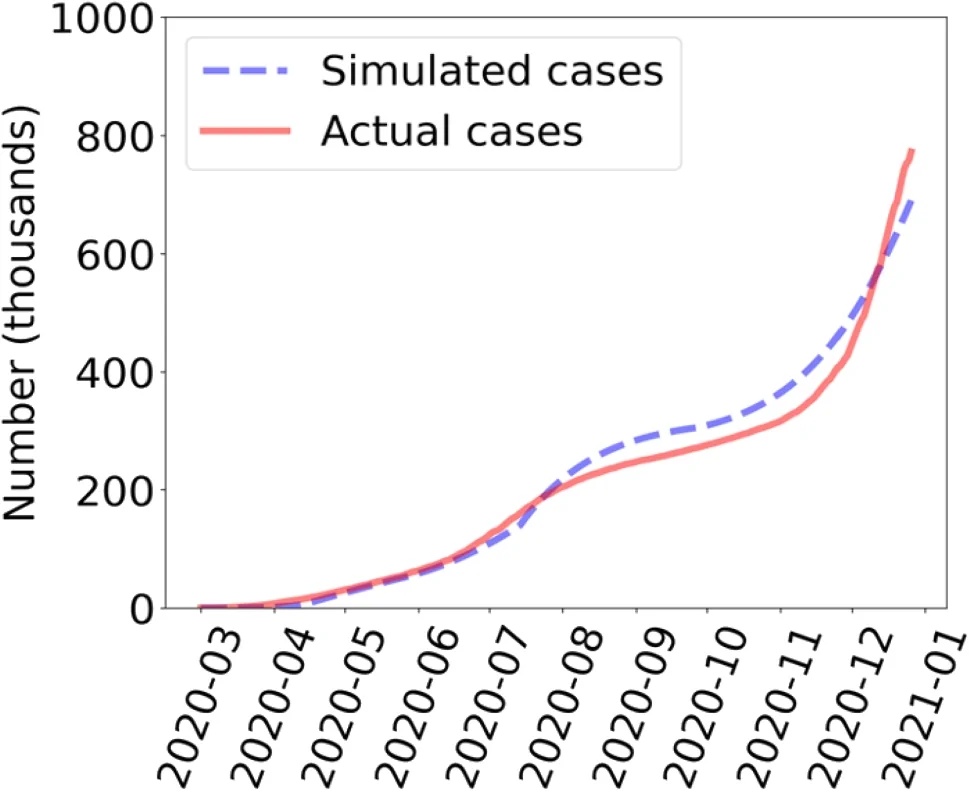
Calibration result of cumulative identified infections on the aggregate level

**Fig. 5:**
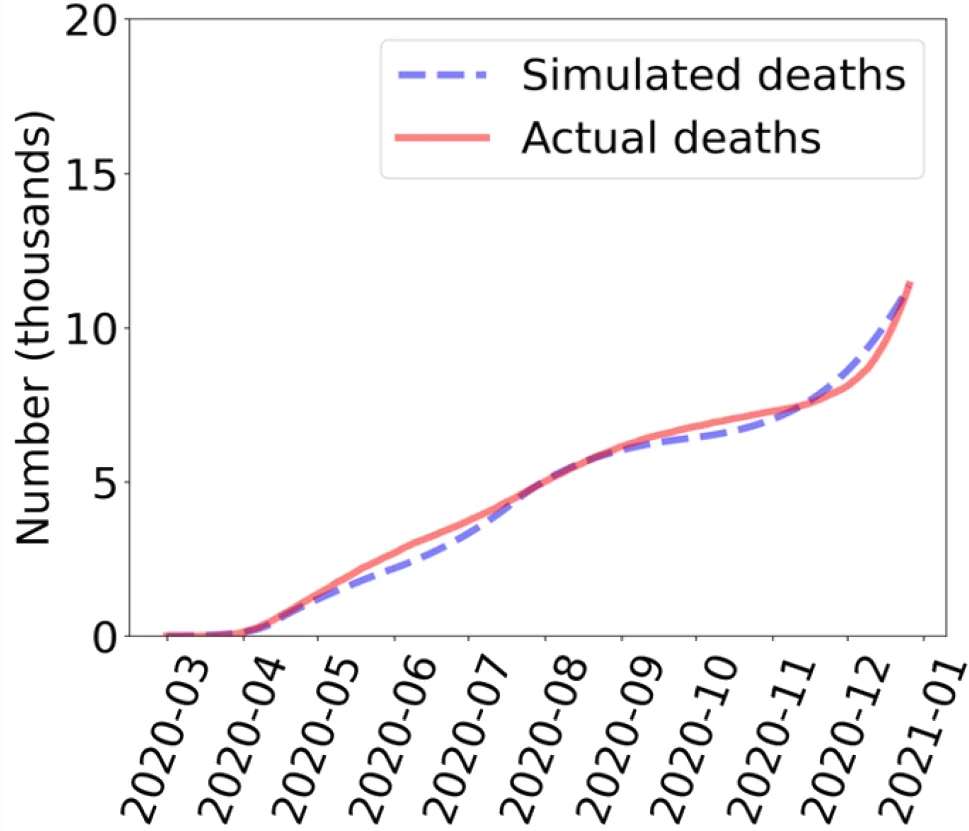
Calibration result of cumulative deaths on the aggregate level

**Fig. 6:**
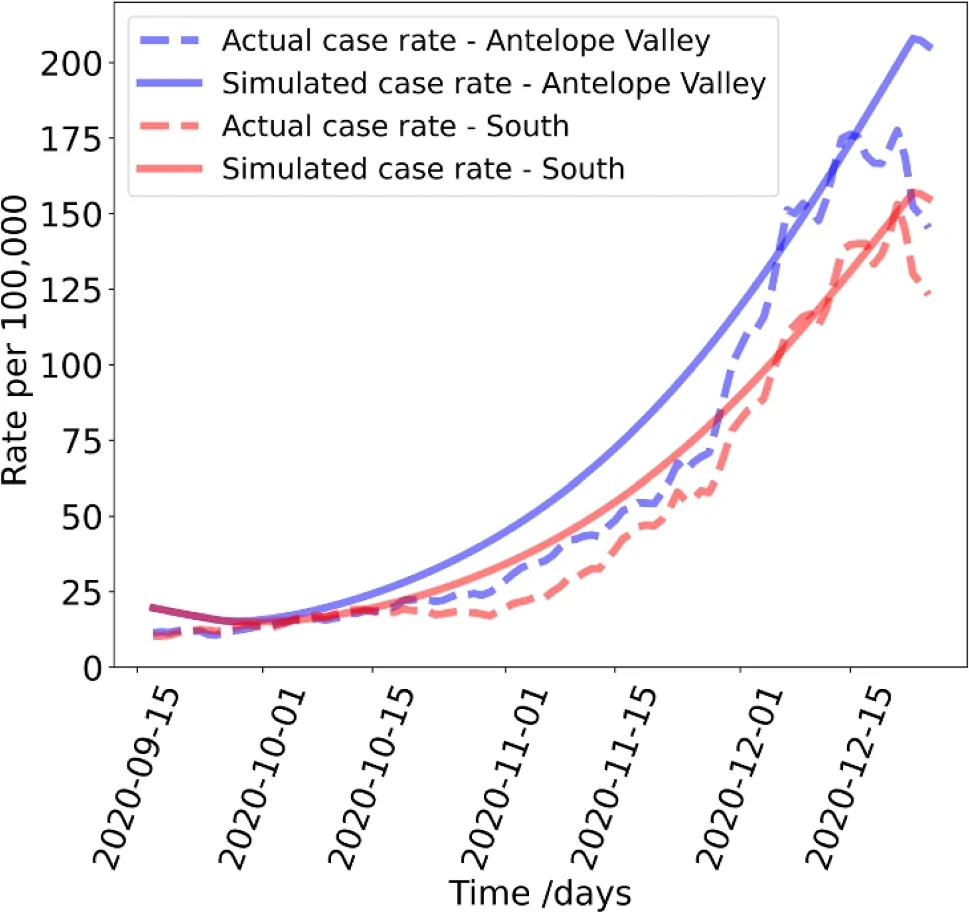
Calibration result of case rate on the HD level

**Fig. 7:**
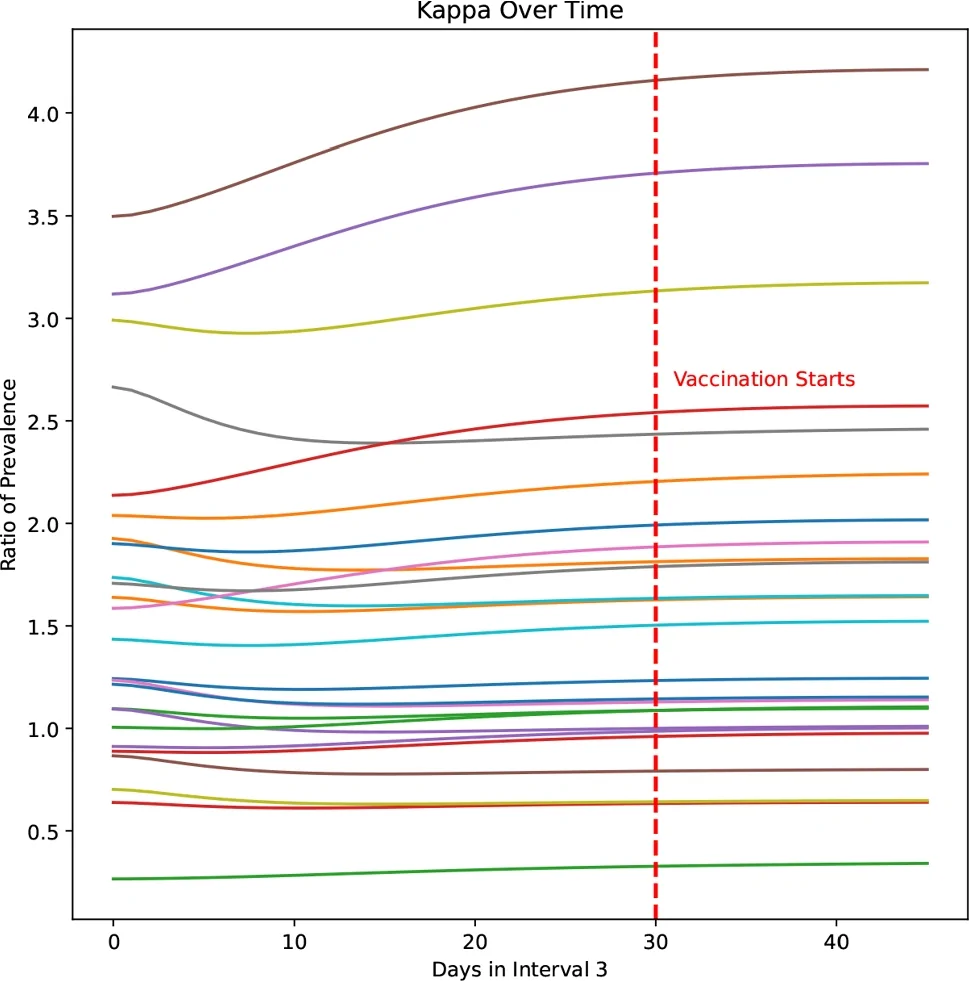
Trajectory of κ over time during the interval of initial vaccination phase

**Fig. 8:**
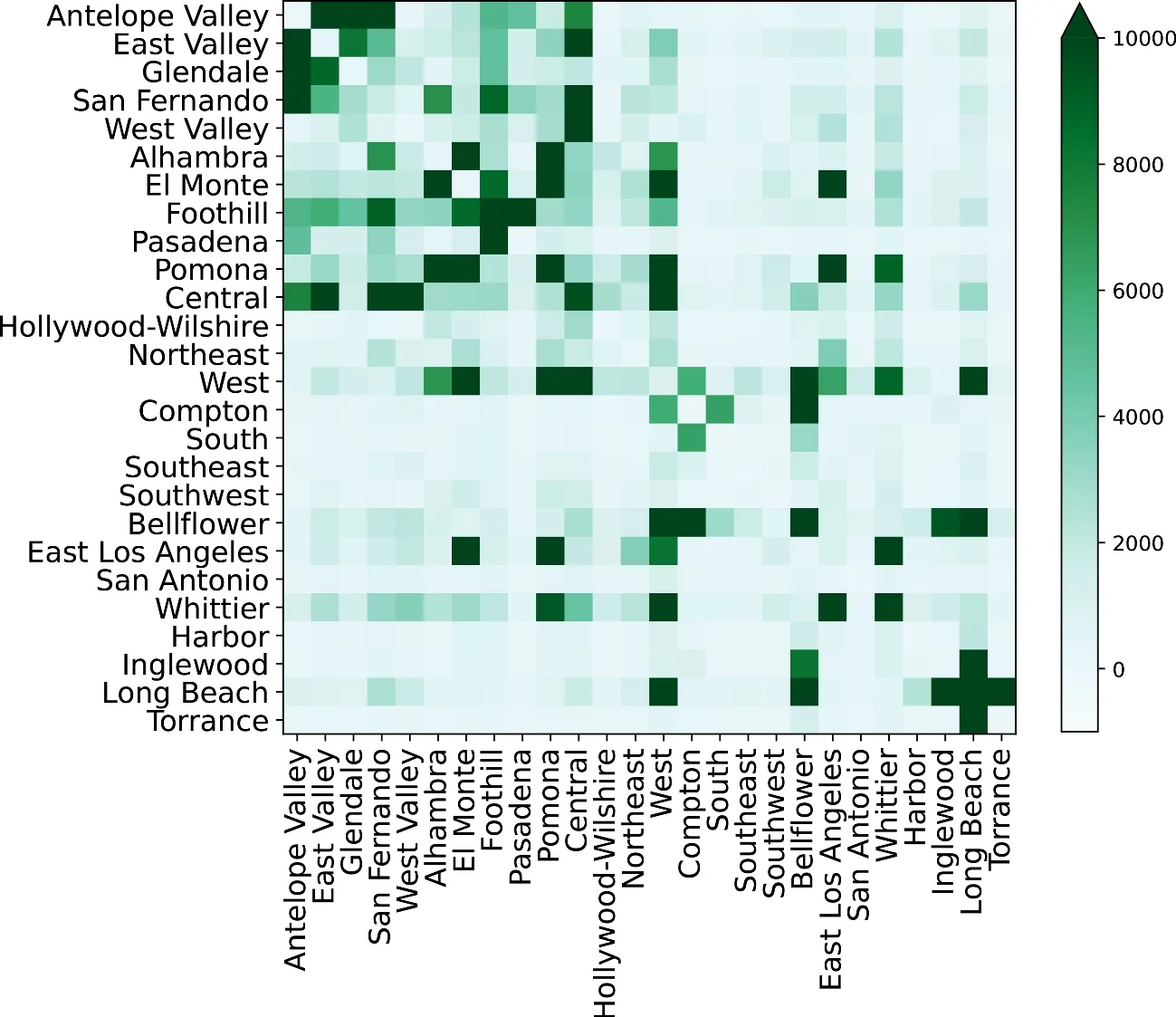
Estimation of daily traffic flow using PeMS data. Each entry represents the number of vehicles going from an origin HD (row) to a destination HD (column). A darker color indicates a higher volume of traffic. We assume individuals live in the origin HD and work in the destination HD

**Fig. 9:**
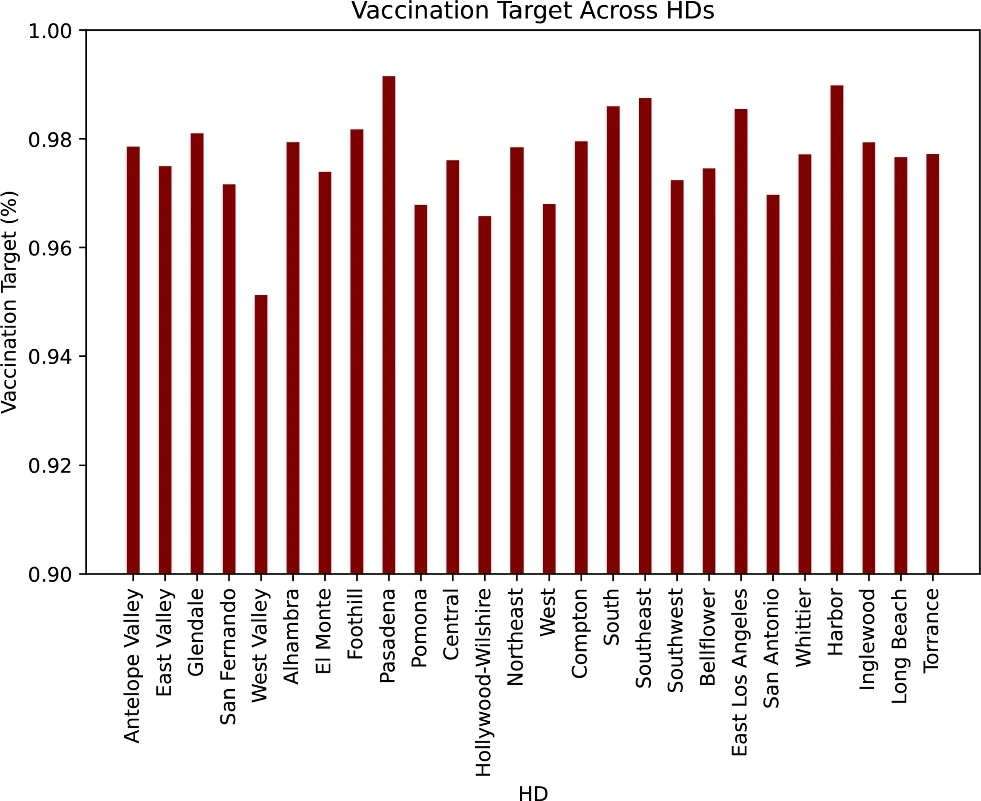
Vaccination targets across HDs

**Fig. 10:**
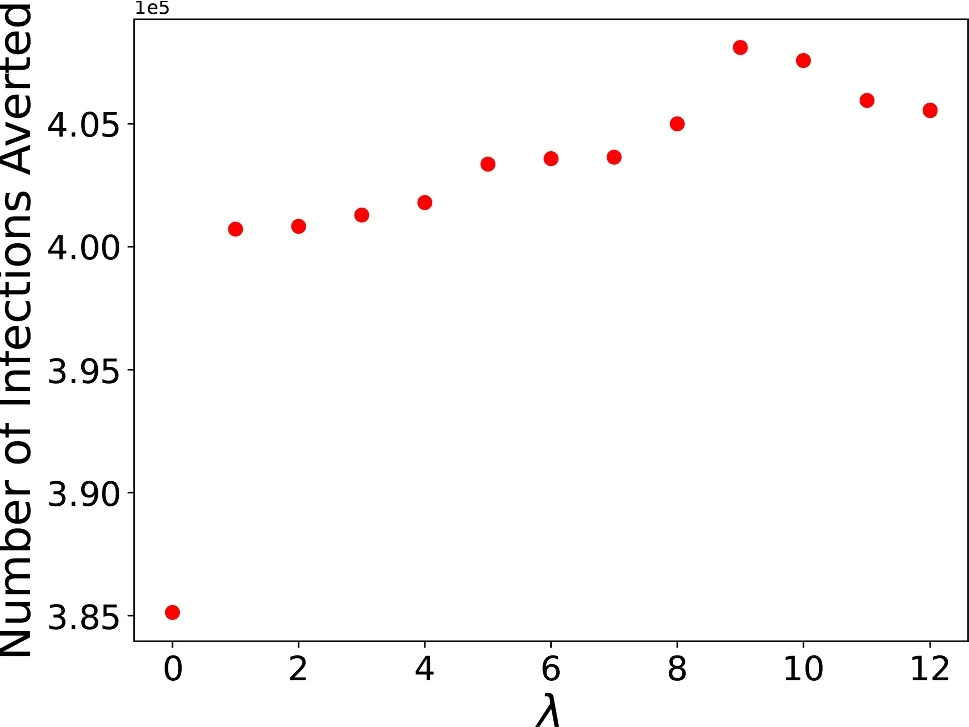
Changes in the number of infections averted with varying λ

**Fig. 11:**
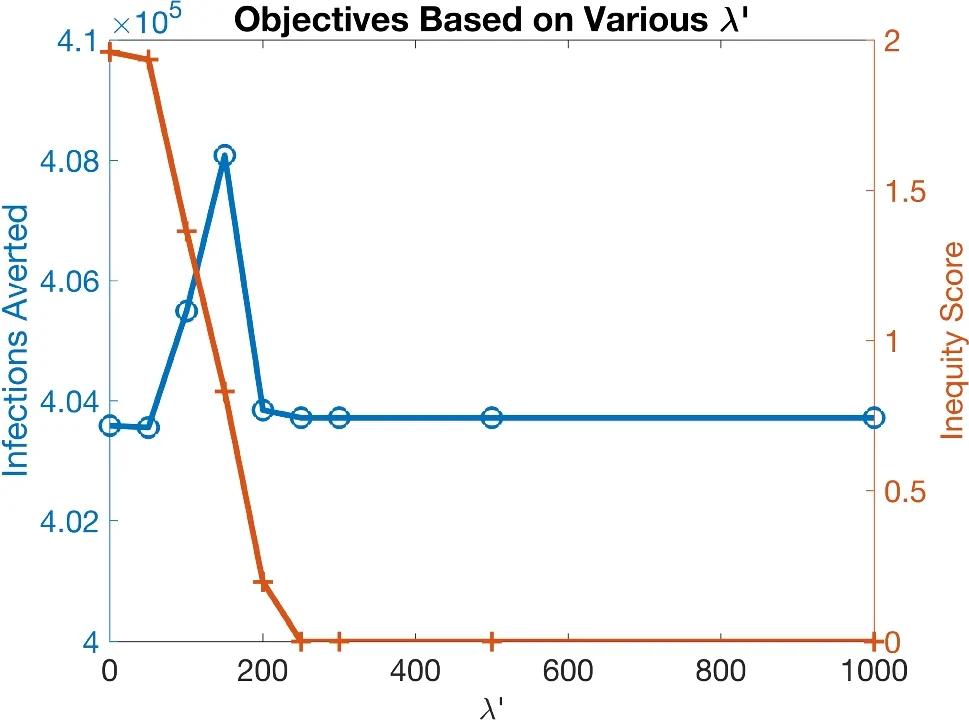
Changes in the number of infections averted and inequity score with varying $\lambda^{\prime }$

## Data Availability

PeMS data can be obtained from Caltrans directly. All other data used in this analysis are publicly available from sources as described in the manuscript.

## Appendix A Compartmental model results

### A.1 Compartmental model dynamics

$$\begin{aligned}&\frac{dS_{v}}{dt}=- \sum _u {\beta _I}_{uv} (t)\times S_v(t) \times \frac{{I}_u(t)}{{N}_u(t)}\\&\quad - \sum _u {\beta _U}_{uv}(t) \times S_v(t) \times \frac{U_u(t)}{{N}_u(t)} - \xi _v (t) \times S_v(t)\\&\frac{dE_v}{dt}= \sum _u {\beta _I}_{uv}(t) \times S_v(t) \times \frac{{I}_u(t)}{{N}_u(t)}\\&\quad + \sum _u {\beta _U}_{uv}(t) \times S_v(t) \times \frac{U_u(t)}{{N}_u(t)}-({\delta _I}(t)+{\delta _U}(t)) \times E_v(t)\\&\frac{dI_v}{dt} = {\delta _I(t)} \times E_v(t)-({\gamma _I(t)}+{\mu _I(t)}+\eta (t)) \times I_v(t)\\&\frac{dU_v}{dt} = {\delta _U}(t) \times E_v(t)-({\gamma _U}(t)+{\mu _U}(t)+\theta (t)) \times U_v(t)\\&\frac{dH_v}{dt} = {\eta }(t) \times I_v(t)+\theta (t)\times U_v(t)-({\gamma _H}(t)+{\mu _H}(t)) \times H_v(t)\\&\frac{dR_v}{dt} = {\gamma _I}(t) \times I_v(t)+{\gamma _U}(t) \times U_v(t)+{\gamma _H}(t) \times H_v(t)\\&\frac{dD_v}{dt} = {\mu _I}(t) \times I_v(t)+{\mu _U}(t) \times U_v(t)+{\mu _H}(t) \times H_v(t)\\&\frac{dV_v}{dt} =\xi _v (t) \times S_v(t) \end{aligned}$$

The presented compartmental model captures the dynamics of a partially vaccinated population in the context of infectious disease transmission (Fig. 3). The population is divided into various compartments: $S_v(t)$ (susceptible individuals), $E_v(t)$ (exposed individuals who are infected but not yet infectious), $I_v(t)$ (identified infected individuals), $U_v(t)$ (unidentified infected individuals), $H_v(t)$ (hospitalized individuals), $R_v(t)$ (recovered individuals), and $D_v(t)$ (dead individuals). Additionally, $V_v(t)$ represents the cumulative number of individuals who have been vaccinated. The model tracks the rates of change for each compartment over time (t) using a set of differential equations. Transmission between individuals is governed by infection rates ($\beta _{Iuv}(t)$ and $\beta _{Uuv}(t)$) based on contact with both identified ($I_u(t)$) and unidentified ($U_u(t)$) infected individuals in the unvaccinated population. Individuals move between compartments due to various processes such as infection, recovery, hospitalization, or death, characterized by rates such as $\delta _I(t)$ (rate of exposed individuals becoming identified infected), $\delta _U(t)$ (rate of exposed individuals becoming unidentified infected), $\gamma _I(t), \gamma _U(t), \gamma _H(t)$ (recovery rates), and $\mu _I(t), \mu _U(t), \mu _H(t)$ (mortality rates). Vaccination dynamics are modeled with a term $\xi _v(t)$, which reduces the number of susceptible individuals by transitioning them into the vaccinated state. $w_D,w_C,w_I,w_H$ are set to 15, 1, 2, and 5.

### A.2 COVID-19 model calibration results

Figure 4 shows the calibration result of cumulative identified infections on the aggregate level. Figure 5 shows the calibration result of cumulative deaths on the aggregate level. Figure 6 shows the calibration result of the case rate on the HD level. All calibration results demonstrate that the calibrated compartmental model is able to capture the disease dynamics in both aggregate and HD levels. Additionally, Fig. 7 shows the trajectory of κ over time during the interval of the initial vaccination phase, supporting the assumption that κ is time-invariant when computing herd immunity thresholds.

## Appendix B Model inputs

### B.1 List of notation

**Table 3:** Table of notation used in P2

	Notation	Definition
Set	$\mathcal {T}_E$	a set of time periods where the health equity objective is considered.
Parameters	$\zeta _u^t$	difference between the vaccination target and vaccinated population for the HD *u* at time *t*.
	$\delta _{u}^t$	prioritization weight for HD *u* at time *t*.
	$\tau _t$	Inequity score at time *t*.

## Appendix C Sensitivity analyses

### C.2 Choices of prioritization scores

**Table 4:** Model results

Model	Travel inconvenience (mins.)	Infections averted	Inequity objective
Betweenness Centrality	210,216,624	405,247	1.799
Population	200,576,766	404,068	0.590
Case rate	201,865,615	401,503	0.447

### C.3 Scenarios of vaccine protection

**Table 5:** Model results

Model	Travel inconvenience (mins.)	Infections averted	Inequity objective
Weekly decisions	203,858,702	405,942	0.779
One month lag protection	202,599,606	341,374	0.411
80% vaccine protection	204,180,382	393,978	0.259

### C.4 Comparison with continuous solution

We compare the difference in *mega-sits*, non-commuter assignments, and commuter assignments between the baseline and continuous solutions. The site assignment remain unchanged when switching to a continuous relaxation. only 1 out of 676 assignment changed with a significant difference of approximately 2500 individuals. Other changes remain small. The overall change is 4 individuals per assignment. For commuters, 1 out of 17,576 has significant change of approximate 400 individuals. The overall change is 0.02 individuals per assignment. Overall, the change in the individual assignment remain small and stable compared with the integer solution. If we additionally considers assignment in all six time periods, out of 105,456 total assignments, only six showed a difference over 10 people. Moreover, due to the rounding process in the continuous version, the total number of vaccinations decreased by 87 (Figs. 8, 9, 10 and 11).
