# Supplementary figures and images for: Optimizing vaccine site locations while considering travel inconvenience and public health outcomes
Source: Health Care Manag Sci. 2026 Jul 13;29(3):30. doi: 10.1007/s10729-026-09778-2 (PMC13364939; doi:10.1007/s10729-026-09778-2)

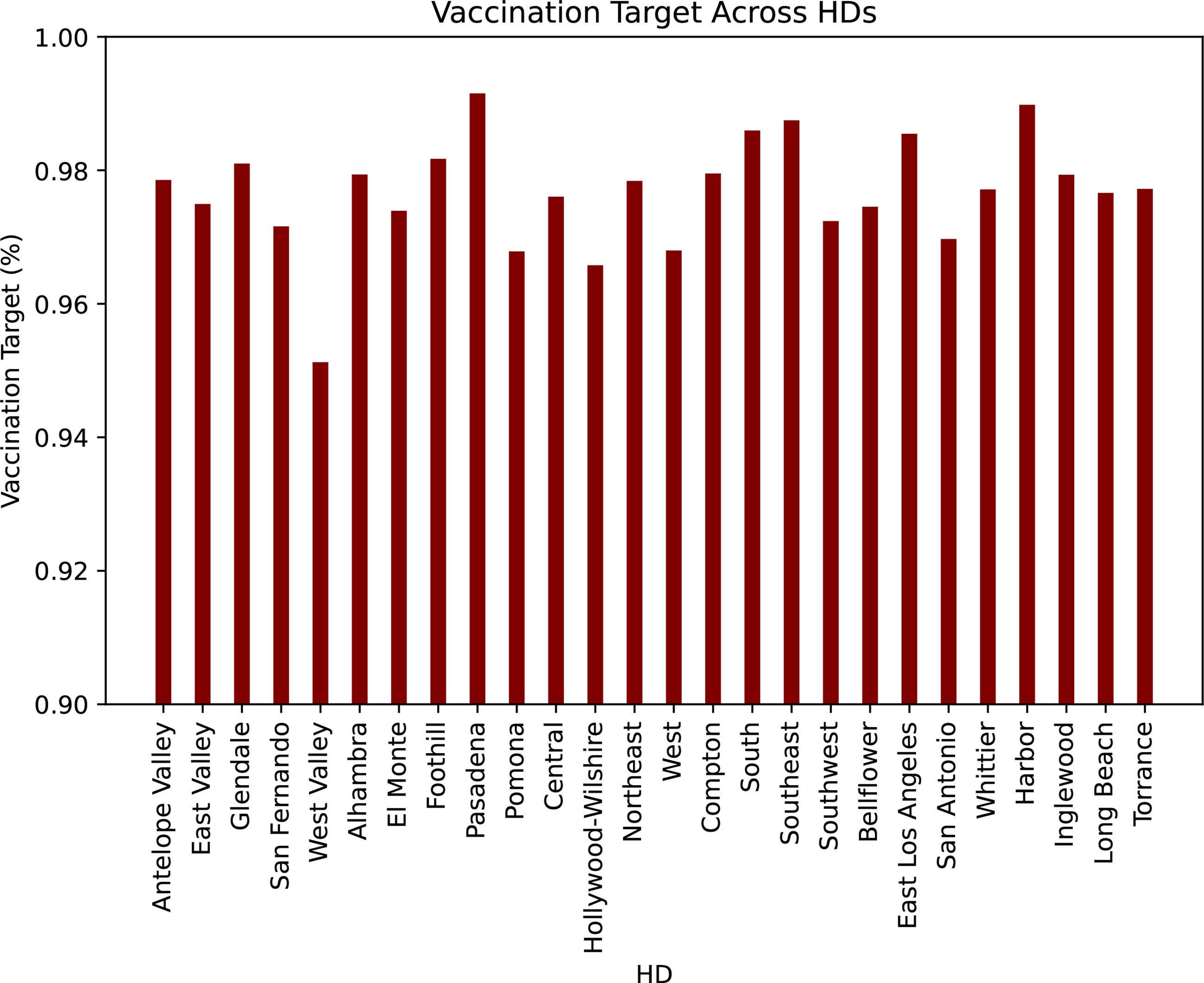

Supplement: Supplementary file 1 — (zip 6.49 MB) [file 10729_2026_9778_MOESM1_ESM.zip › fmc9.tif]

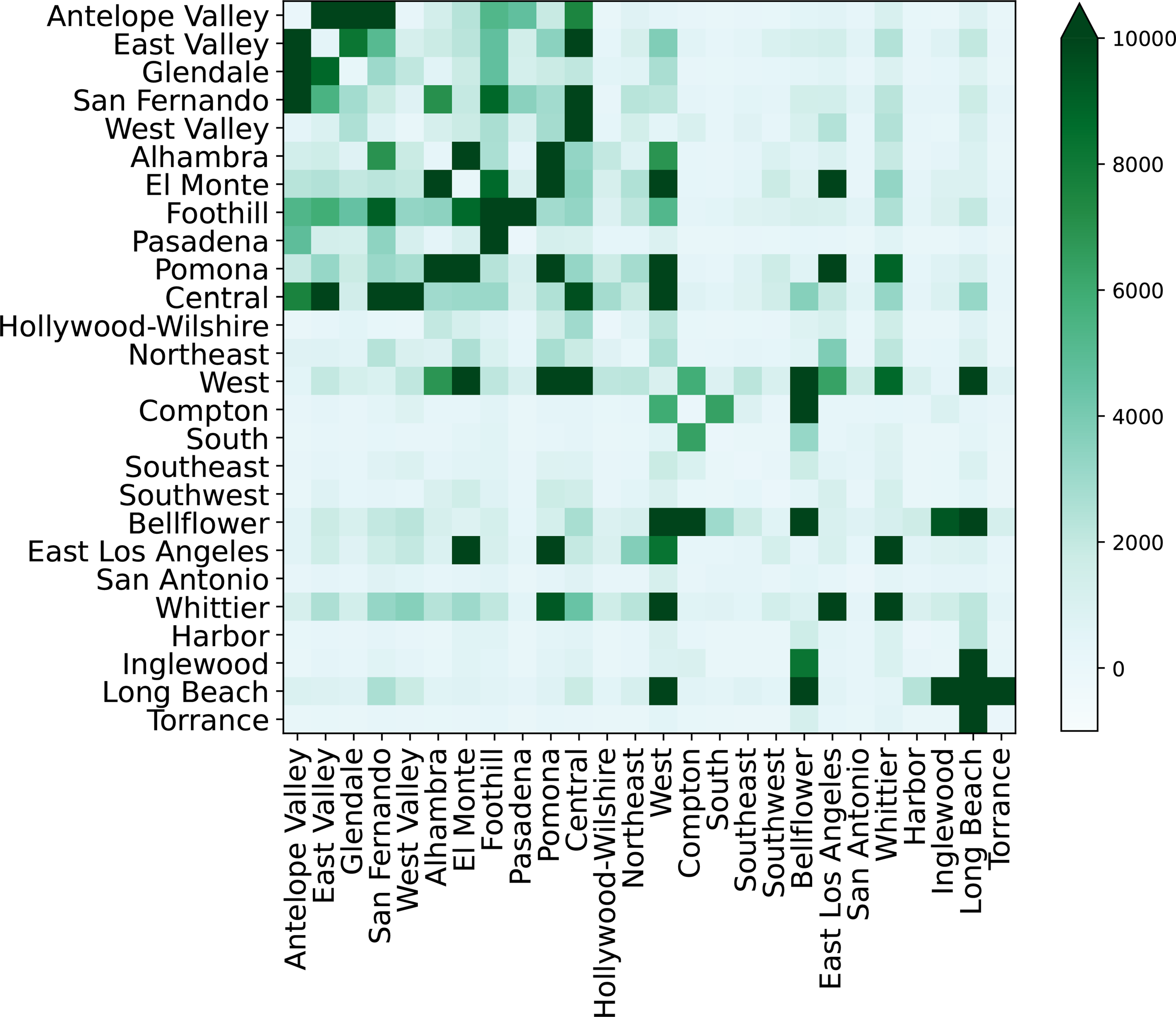

Supplement: Supplementary file 1 — (zip 6.49 MB) [file 10729_2026_9778_MOESM1_ESM.zip › fmc8.tif]

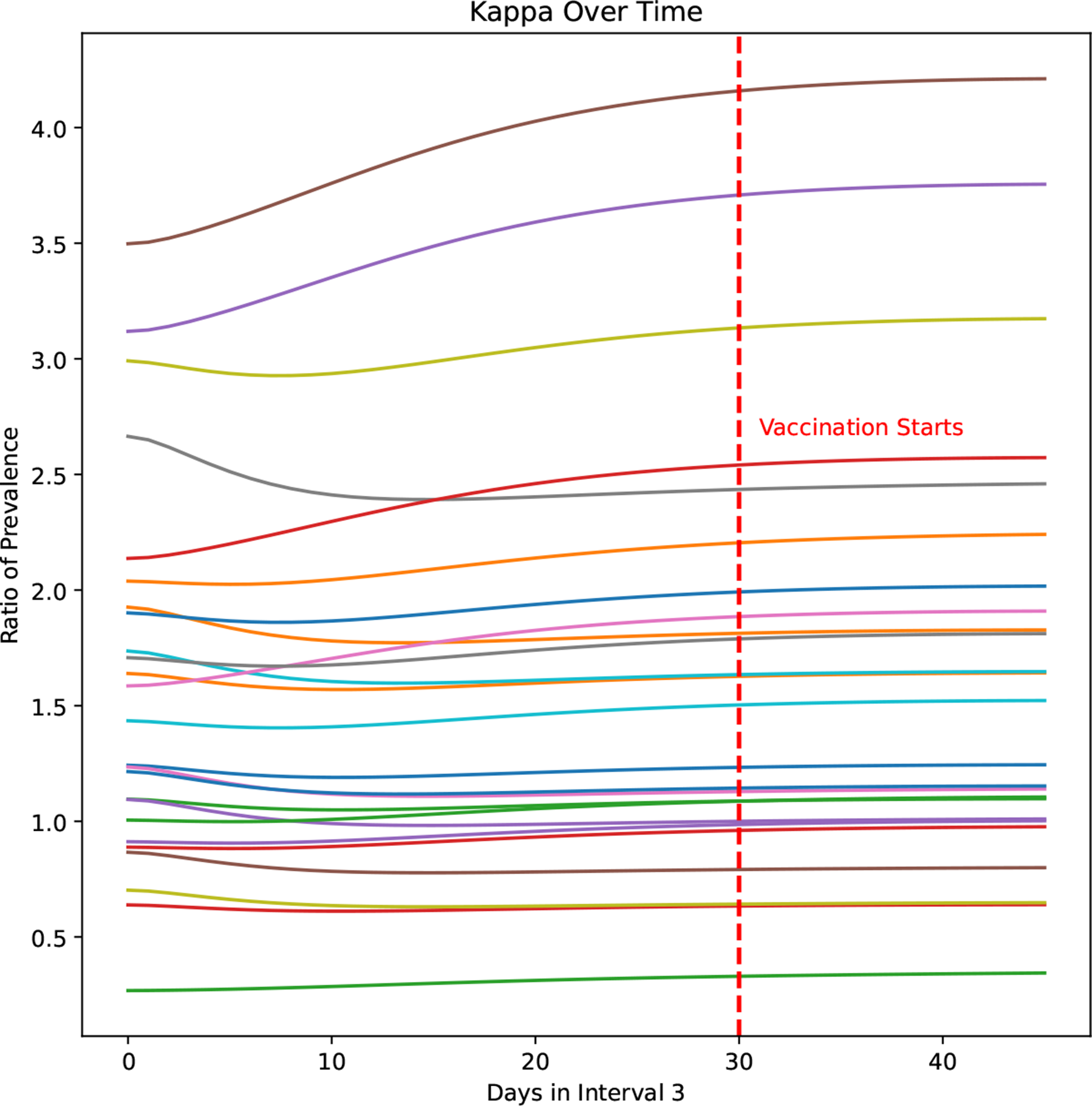

Supplement: Supplementary file 1 — (zip 6.49 MB) [file 10729_2026_9778_MOESM1_ESM.zip › fmc7.tif]

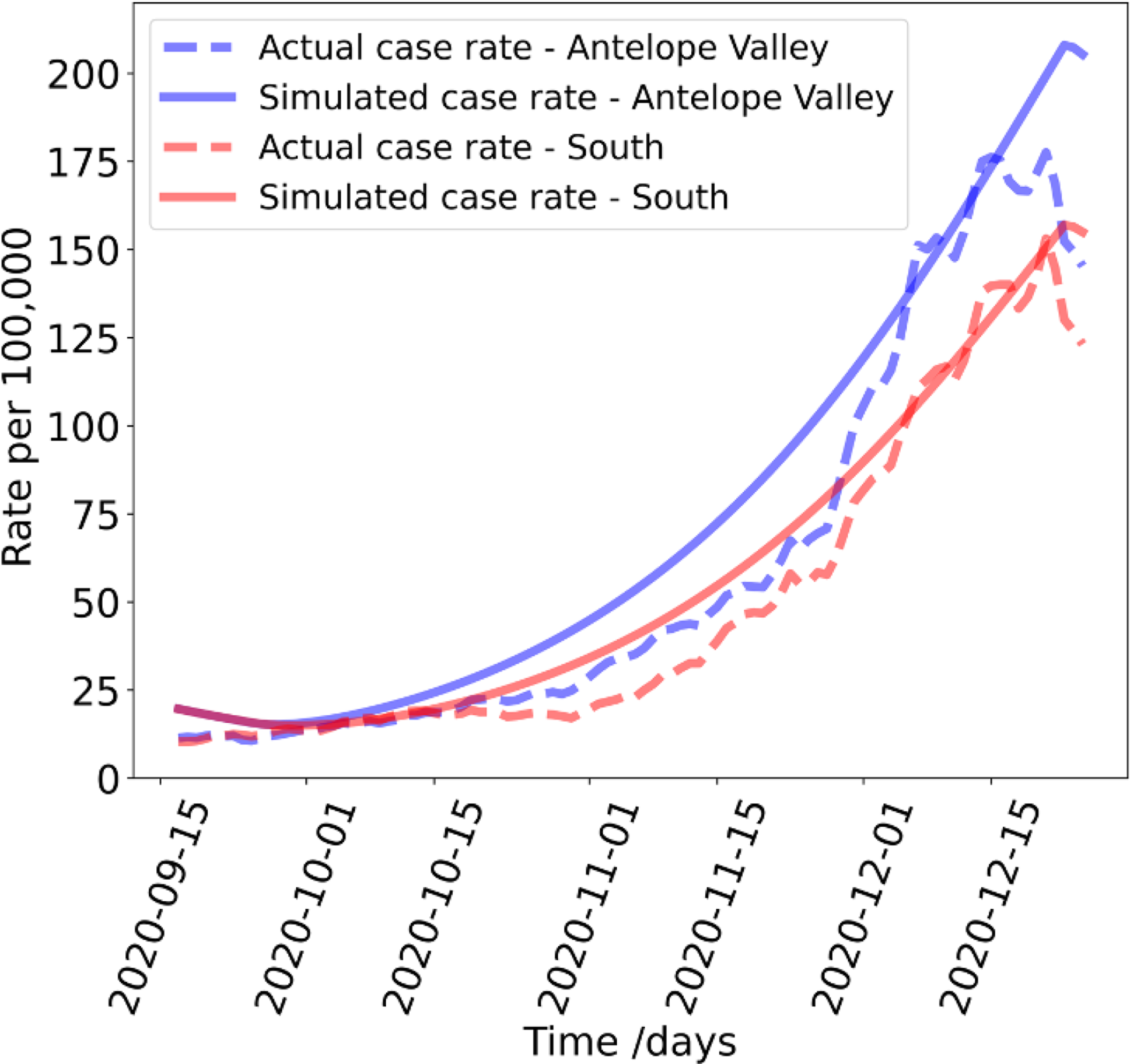

Supplement: Supplementary file 1 — (zip 6.49 MB) [file 10729_2026_9778_MOESM1_ESM.zip › fmc6.tif]

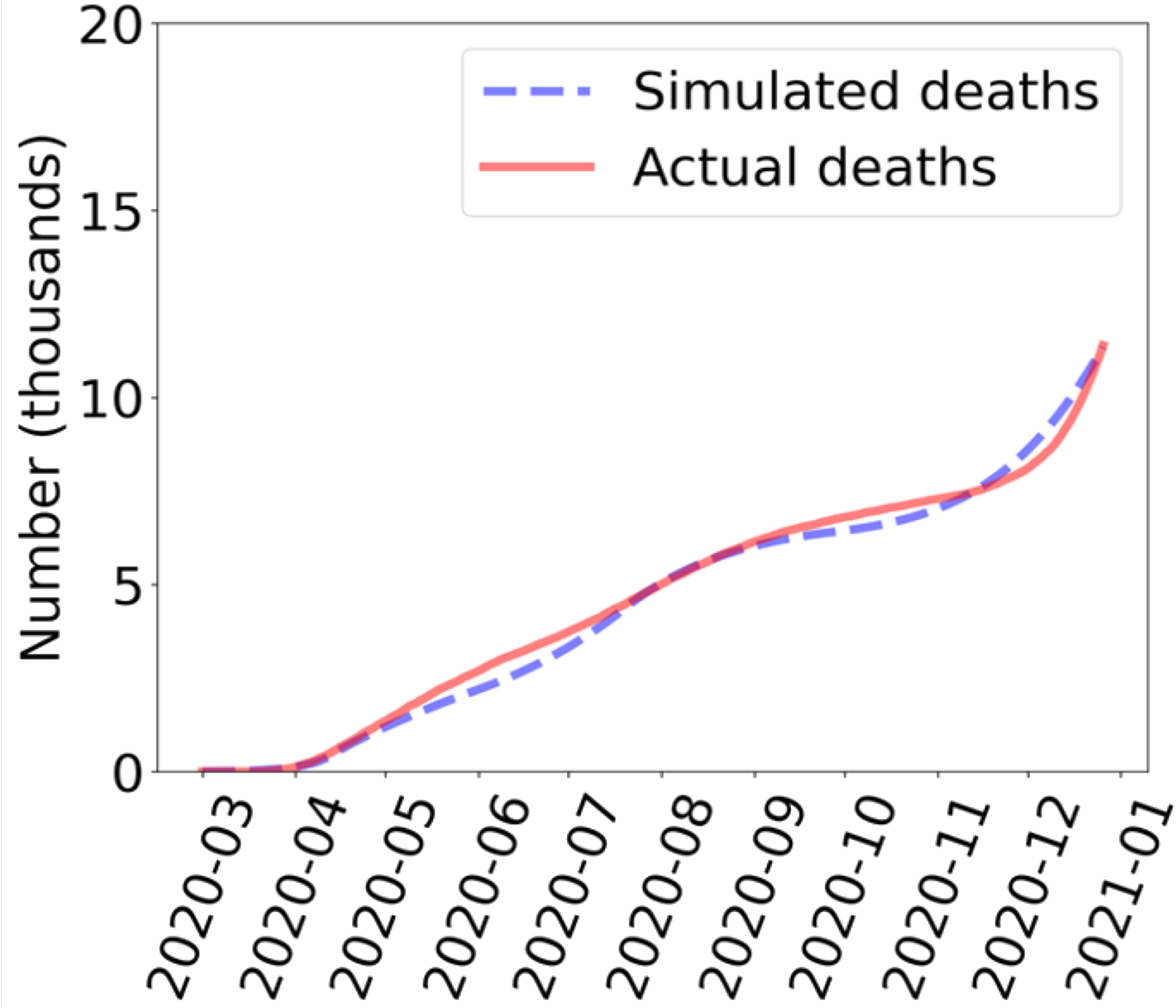

Supplement: Supplementary file 1 — (zip 6.49 MB) [file 10729_2026_9778_MOESM1_ESM.zip › fmc5.tif]

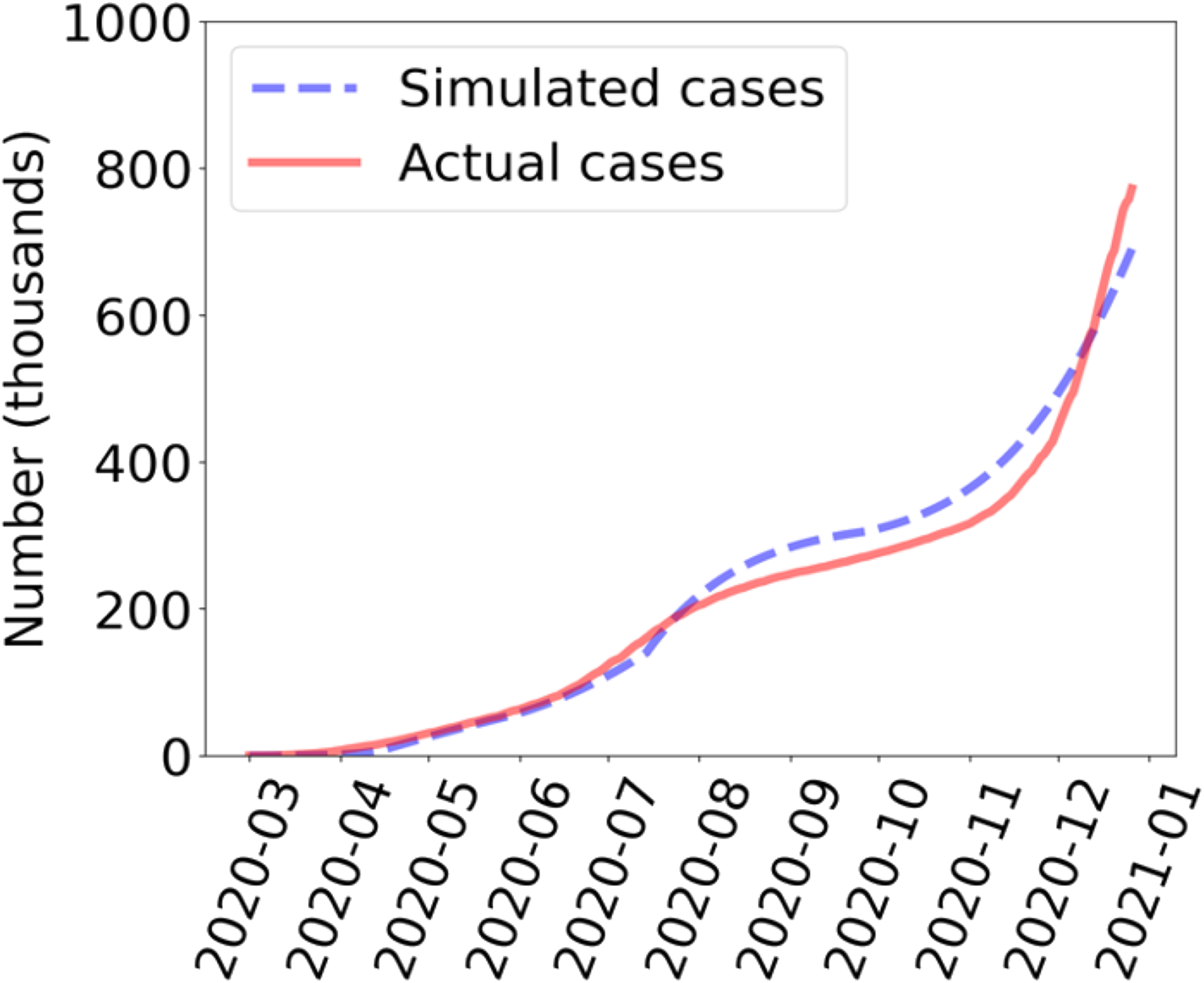

Supplement: Supplementary file 1 — (zip 6.49 MB) [file 10729_2026_9778_MOESM1_ESM.zip › fmc4.tif]

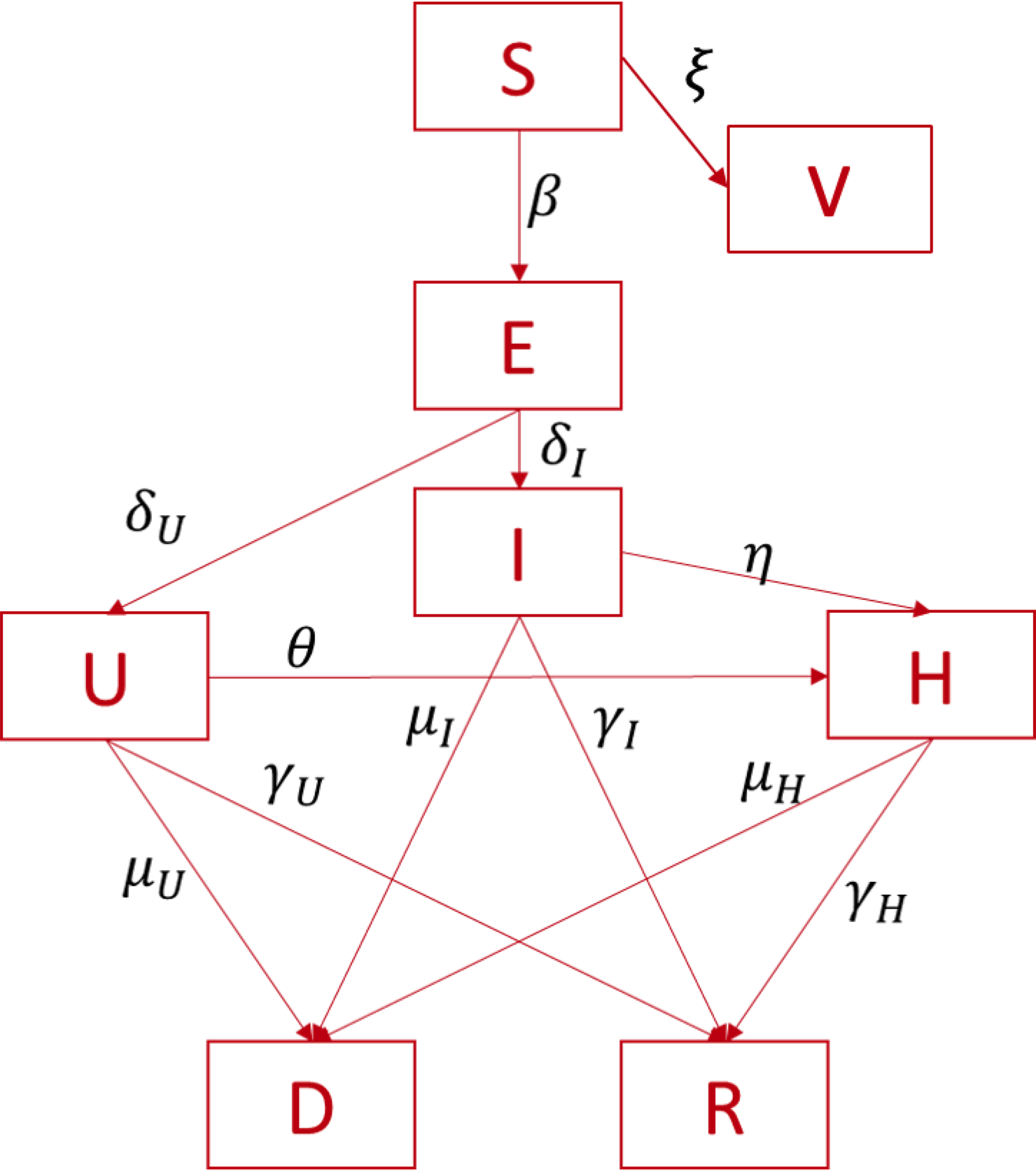

Supplement: Supplementary file 1 — (zip 6.49 MB) [file 10729_2026_9778_MOESM1_ESM.zip › fmc3.tif]

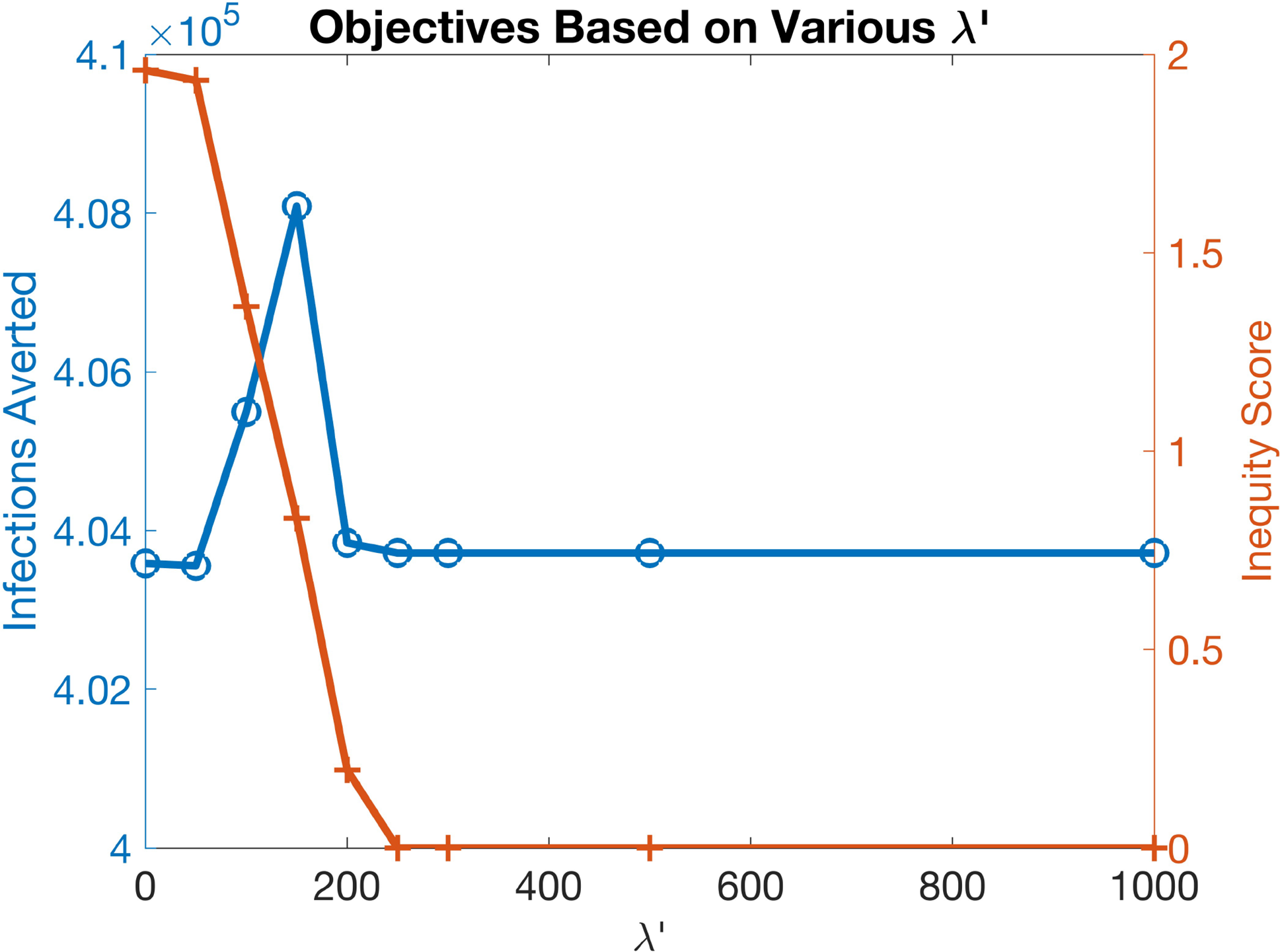

Supplement: Supplementary file 1 — (zip 6.49 MB) [file 10729_2026_9778_MOESM1_ESM.zip › fmc11.tif]

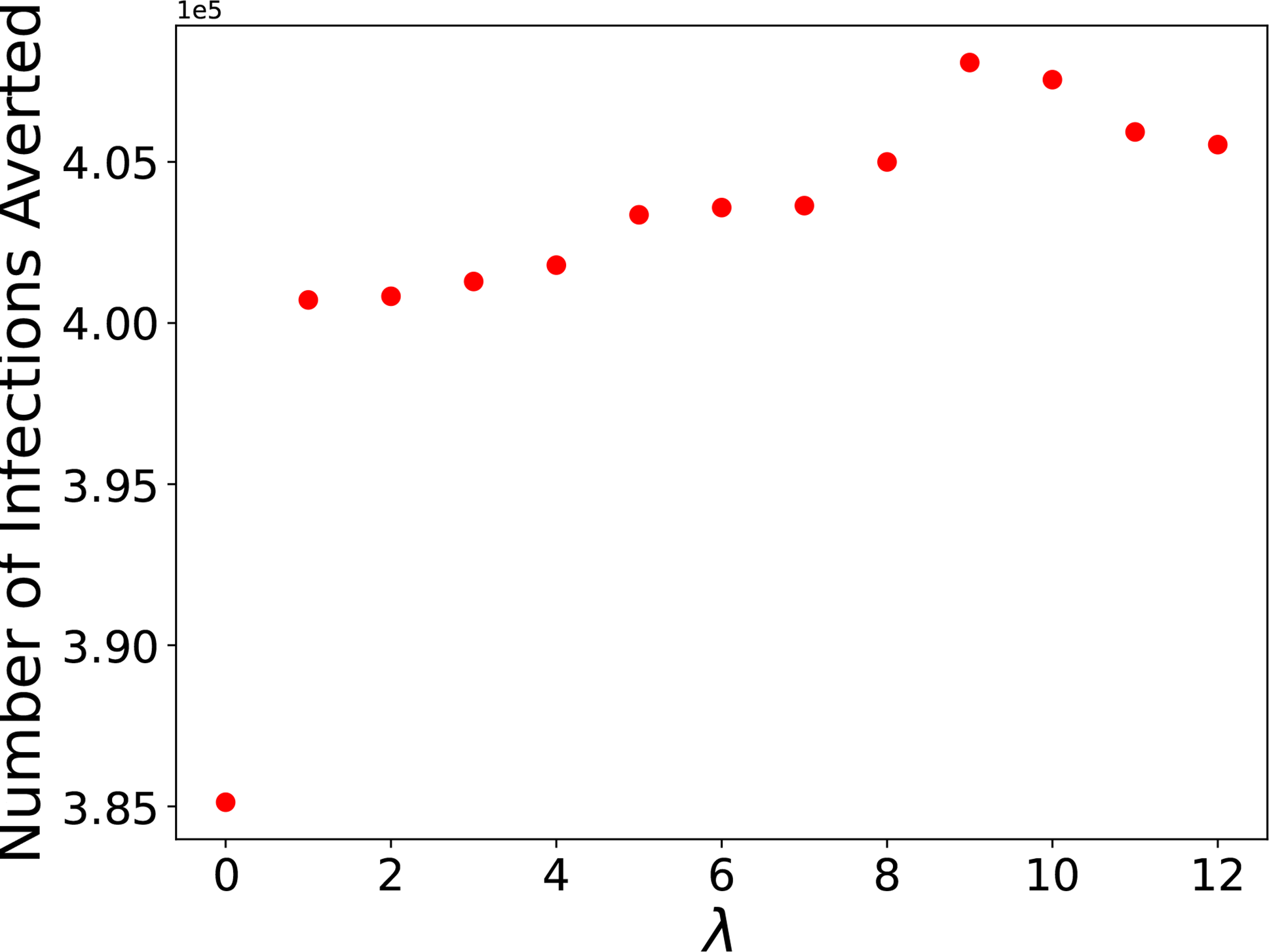

Supplement: Supplementary file 1 — (zip 6.49 MB) [file 10729_2026_9778_MOESM1_ESM.zip › fmc10.tif]

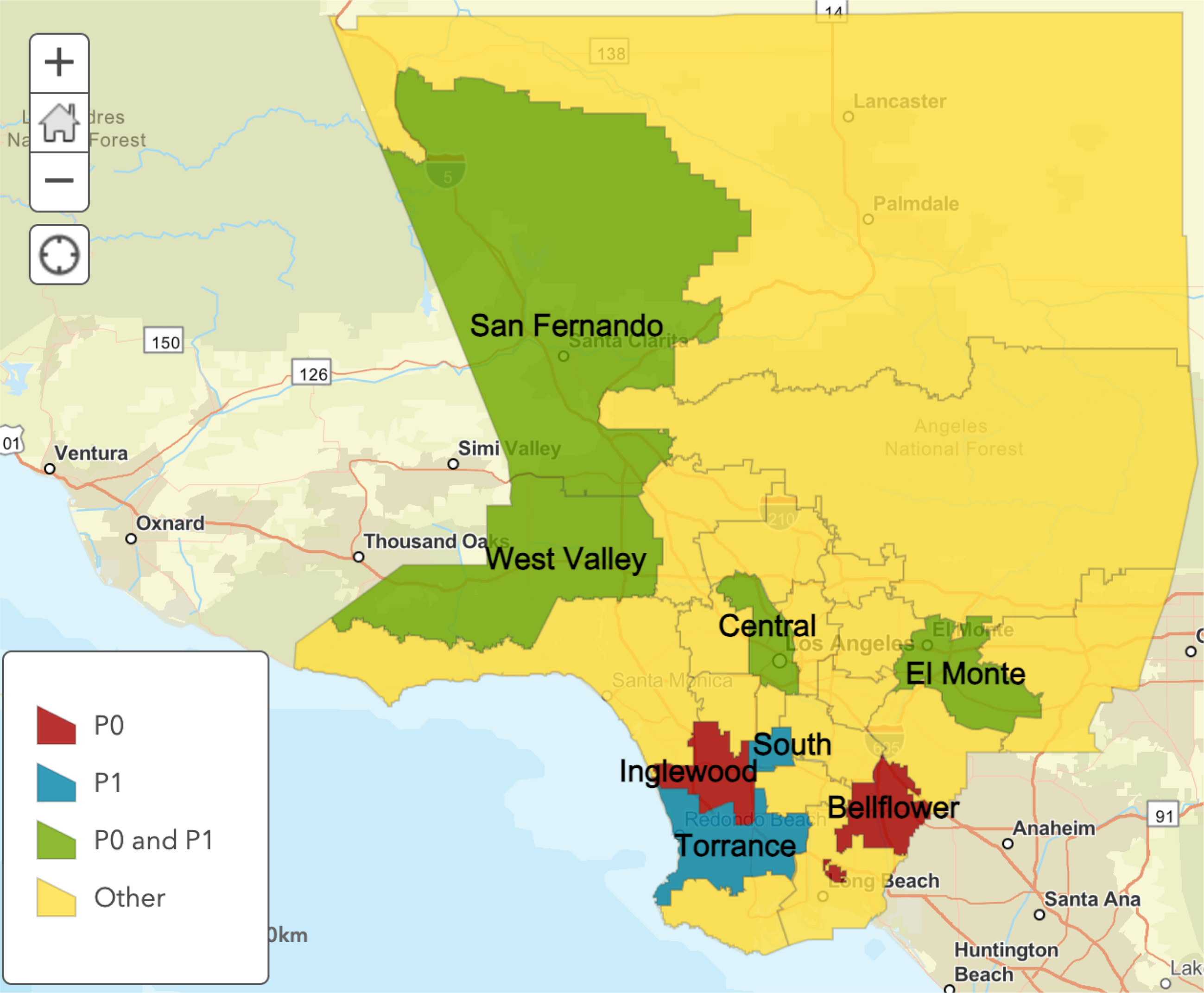

Supplement: Supplementary file 1 — (zip 6.49 MB) [file 10729_2026_9778_MOESM1_ESM.zip › fmc1.tif]
